# Bioinformatic characterization of endolysins and holin-like membrane proteins in the lysis cassette of phages that infect *Gordonia rubripertincta*

**DOI:** 10.1371/journal.pone.0276603

**Published:** 2022-11-17

**Authors:** Richard S. Pollenz, Jackson Bland, Welkin H. Pope

**Affiliations:** 1 Department of Cell Biology, Microbiology and Molecular Biology, University of South Florida, Tampa, Florida, United States of America; 2 Science Department, Chatham University, Pittsburgh, Pennsylvania, United States of America; Roskilde Universitet, DENMARK

## Abstract

Holins are bacteriophage-encoded transmembrane proteins that function to control the timing of bacterial lysis event, assist with the destabilization of the membrane proton motive force and in some models, generate large “pores” in the cell membrane to allow the exit of the phage-encoded endolysin so they can access the peptidoglycan components of the cell wall. The lysis mechanism has been rigorously evaluated through biochemical and genetic studies in very few phages, and the results indicate that phages utilize endolysins, holins and accessory proteins to the outer membrane to achieve cell lysis through several distinct operational models. This observation suggests the possibility that phages may evolve novel variations of how the lysis proteins functionally interact in an effort to improve fitness or evade host defenses. To begin to address this hypothesis, the current study utilized a comprehensive bioinformatic approach to systematically identify the proteins encoded by the genes within the lysis cassettes in 16 genetically diverse phages that infect the Gram-positive *Gordonia rubripertincta* NRLL B-16540 strain. The results show that there is a high level of diversity of the various lysis genes and 16 different genome organizations of the putative lysis cassette, many which have never been described. Thirty-four different genes encoding holin-like proteins were identified as well as a potential holin-major capsid fusion protein. The holin-like proteins contained between 1–4 transmembrane helices, were not shared to a high degree amongst the different phages and are present in the lysis cassette in a wide range of combinations of up to 4 genes in which none are duplicated. Detailed evaluation of the transmembrane domains and predicted membrane topologies of the holin-like proteins show that many have novel structures that have not been previously characterized. These results provide compelling support that there are novel operational lysis models yet to be discovered.

## Introduction

### Phage bacterial lysis proteins

Bacteriophages are viruses that infect bacteria and it is estimated that due to the estimated 10^31^ phage particles in the world, phage lysis of bacterial hosts is predicted to be one of the most common biological mechanisms [1]. The use of phages in health has taken on renewed interest due to their potential use as natural antibiotics to combat antibiotic resistant bacteria [reviewed in 2, 3]. In addition, phages are being used in food science and environmental applications to target pathogenic strains [reviewed in: 4, 5]. Although the ability of a phage to lyse their target bacterial host is essential to their utility in all of these applications, the study of phage lysis is limited to very few bacterial hosts. Elegant biochemical and genetic studies on dsDNA phages that infect Gram-negative bacteria have established the key proteins involved in the lysis pathway and defined several different models for how they initiate and complete lysis [see several excellent reviews in this area: 6, 7]. However, it is unclear if these models apply to all phages and the lysis proteins and pathways for phages that infect Gram-positive bacteria remain mostly unexplored at the biochemical level. The impetus to better understand the scope of phage lysis pathways is also underscored by work showing that bacteria may be evolving host defense mechanisms that target the lysis pathway and occur after a productive phage infection [8].

The lytic phage lifecycle of dsDNA phages involves infection, replication, virion assembly and genome packaging, and then cell lysis. In phages that infect Gram-negative bacteria, phages must overcome the inner membrane (IM), peptidoglycan layer (PG) and the outer membrane (OM) to achieve efficient release of phage progeny. Extensive biochemical and genetic work has established a three-step model for the lysis pathway that involves three distinct sets of proteins. The first are endolysin/s that have defined catalytic activity to lyse the PG layer [9]. The second are small proteins with 1–4 transmembrane helices (TM), that function as a holin or antiholin. These proteins work together to, 1) control the timing of when the lysis event will be triggered after phage infection [10, 11], 2) assist with the destabilization of the membrane proton motive force [10, 11] and 3) in some models, generate large “pores” in the cell membrane to allow the exit of the endolysin enzymes so they can access the PG components [12, 13]. Finally, there are membrane proteins and lipoproteins termed spanins that are secreted prior to lysis and serve to fuse the inner (IM) and outer membrane (OM) after PG degradation to allow the phage progeny to be released [14–16].

### Phage bacterial lysis models in Gram-negative hosts

**Fig 1:**
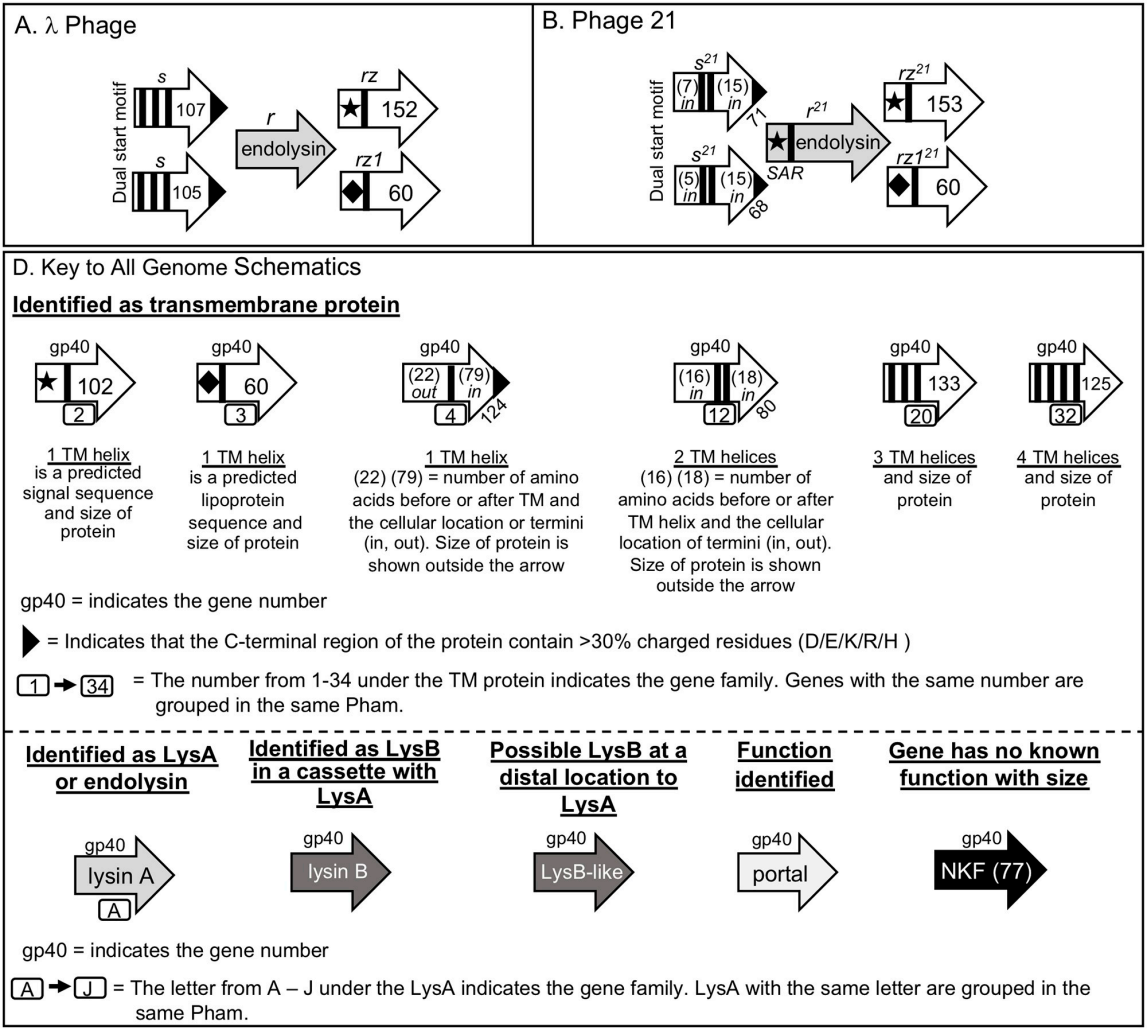
Proteins within the lysis cassettes of phages that infect Gram negative bacteria and key to genome schematics. Panel A: the lysis cassette proteins from λ phage. Panel B: the lysis cassette proteins from phage 21. The direction of transcription of the gene is represented by the arrowhead. The identity of each protein is shown above the arrow. Panel D. The key to all of the genome organization schematics within this report. Note that the Pham designation for the LysA and holin-like genes is represented by a letter or number so that proteins that are grouped together can be readily identified. The corresponding genes can also be found in Tables 2 and 5.

Currently, there are two types of models for how the endolysins, holins/antiholins and spanins interact to complete the lysis pathway in Gram-negative bacteria [6, 7]. The first model is typified by λ phage. In λ, the lysis cassette is in a direct linear gene arrangement with: 1) the holin/antiholin *S* gene that encodes either the 105 amino acid holin or 107 amino acid antiholin with three transmembrane domains using a dual start motif; 2) an endolysin (*R* gene) and 3) two overlapping spanin ORFs that generate a large protein (Rz) with an N-terminal TM domain and a small protein (Rz1) with a lipid association sequence that allows its attachment to the OM (Fig 1 panel A). After phage infection, all lysis proteins accumulate harmlessly in the cell with the holin and antiholin forming both inactive heterodimers (holin-antoholin) and active homodimers (holin-holin) in the IM. At a point in time after infection, the active homodimers reach a critical concentration resulting in oligomerization and the formation of larger protein rafts that lead to the collapse of the proton motive force (PMF). This triggering event and loss of PMF allows the antiholin to become active through insertion of all 3 TM regions in the membrane and with the holin, generate concatemers that create large enough holes in the IM to allow exit of the folded endolysin to PG layer. Once the PG is destroyed, the spanins become activated to fuse the IM and OM so that lysis is complete. Amazingly, the time from triggering to lysis is instantaneous, not gradual [15]. Phage P2 has a similar linear gene arrangement and appears to utilize the same general mechanism to λ except that this phage does not use the dual start mechanism to produce the antiholin gene from gene *Y* but encodes the antiholin protein, LysA, from a separate gene immediately downstream of the endolysin gene *K* [6, 7].

The second pathway, typified by the phage 21 [6, 7], has the exact same gene organization as λ, with the holin (S^21^68) and antiholin (S^21^71) produced with a dual start motif (Fig 1 panel B). These proteins however, only have 2 TM helices in comparison to the 3 TM helices of the λ holin proteins. As with λ, both types of holin accumulate in the membrane in active and inactive complexes. Upon triggering, the N-terminal TM domains of both holins become extracellular and the proteins form homopentamers through the interaction of the embedded TM helices. These homopentamers do not create large holes like the holins in λ but generate up to 10^3^ 2nm “pinholes” in the IM. In this model, the size of the pinholes is not sufficient to allow the exit of the native endolysin and thus, the endolysin is secreted from the cell prior to triggering through an N-terminal TM helix that also serves as a signal-arrest-release sequence (SAR) to keep the enzyme attached to the membrane in an inactive state prior to the triggering event [17, 18]. The loss of the PMF releases the endolysin that now folds into an active form. Thus, in both lysis models, the same general set of proteins are utilized for the lysis pathway and aside from the phage P2, contain only one endolysin and one holin gene.

### Phage bacterial lysis models in Gram-positive hosts

The model for the lysis by phages that infect Gram-positive bacteria is not as well studied and generally restricted to phages that infect actinobacteria. Phage lysis in Gram-positive bacteria must overcome the IM and PG, but Gram-positive bacteria generally lack a formal OM and instead have a much more complex PG layer and often also contain extended structural cell wall components beyond the PG later such as arabinogalactans and mycolic acids [19–21]. Phages that infect Gram positive bacteria are expected to utilize a similar set of endolysin and holins to overcome these barriers. Indeed, a diversity of endolysins with multiple activities against the PG [22–26], a second endolysisn termed lysin B that targets the mycolic acids [24, 27–29] and TM proteins that might serve as holins have been identified in phage genomes that infect Gram positive bacteria [24, 27, 30]. However, how these proteins interact and function during the lysis pathway has been biochemically and genetically evaluated in very few phages.

Phage Ms6 infects *M*. *smegmatis* and has a linear five gene arrangement for the putative lysis cassette that contains, 1) endolysin or lysin A (gp2), 2) lysin B (gp3), 3) gp4 that encodes a small 77 amino acid protein containing two predicted TM helices and 4) gp5 that encodes a protein containing a single TM helix [31–33, reviewed 23]. Several studies suggest that both gp4 and gp5 work together to provide holin function and lysis timing since a null mutant of gp4 does not fully block phage lysis but results in reduced lysis time, whereas mutation of gp5 results in a delay in lysis timing [31, 33]. Cross-linking studies also allow recovery of gp4-gp5 complexes suggesting that the 2 proteins interact with each other [31]. However, these holins are not sufficient for the release of the lysin A out of the cell and appear to be functioning more like pinholins of phage 21 [31–33]. Since the full length Ms6 lysin A does not have a SAR domain similar to the endolysin from phage 21, it was determined that a novel protein expressed from gp1 (directly upstream of the lysin A), serves as a chaperone to assist with the transport the lysin A from the cell by using the bacterial secretory system [33]. The mechanism for the release of the lysin B from phage Ms6 is still unresolved, but is proposed to occur through simple diffusion after triggering and the destruction of the PG layer by the lysin A [23]. Aside from gene deletion studies that identify endolysins and holin-like genes in phage Giles as essential for phage propagation [30], there are few other studies of phages that infect Gram-positive hosts that have rigorously evaluated the phage lysis pathway to the extent of the work with λ and the other E. coli phages. Thus, the functional operation of the lysis pathway in Ms6, represents yet a third model that is distinct from the two that have been defined for the phages that infect Gram-negative bacteria.

It is striking that in the phages where the lysis mechanism has been rigorously evaluated through biochemical and genetic studies, that each phage would utilize endolysins, holins and accessory proteins to the OM to achieve cell lysis through three distinct operational models. This observation suggests the possibility that other phages may evolve novel variations of how the lysis proteins functionally interact in an effort to improve fitness or evade host defenses. A first step in addressing this hypothesis is to carry out a comprehensive analyses of the proteins involved in the lysis pathway of diverse phages to assess the genomic diversity of the lysis cassettes. The identification of endolysins in phages can help establish the location of the putative lysis cassette and there are numerous crystal structures and functional domains that can be utilized to identify these proteins from the various protein/DNA repositories. The holins on the other hand, do not share amino acid identity, do not have conserved functional domains and in many genomes are only annotated if a gene that encodes a protein with at least 2 TM domains is found in association with a lysA [6, 7, 23–25]. Thus, many holins may not have been annotated properly or been left un-annotated. It is necessary therefore, to identify the diversity of holin-like genes by completing a rigorous gene-by-gene analysis of individual phage genomes.

### Phages that infect *Gordonia rupbripertincta*

The current study utilized a comprehensive bioinformatic approach to systematically identify the proteins encoded by the genes within the lysis cassettes in a set of 77 diverse phages that infect the Gram-positive *Gordonia rubripertincta* NRLL B-16540 strain. *Gordonia sp*. are members of the Actinobacteria, commonly found in soil [34]. Various species have been implicated in foaming of wastewater in treatment plants (*G*. *terrae)* [35], catheter infections or other infections in immunocompromised patients (*G*.*terrae*, *G*.*bronchalis)* [36–38] and as a causative agent in bovine mastitis (*G*. *paraffinivorans)* [39]. *Gordonia sp*. are from the same lineage as *Mycobacterium tuberculosis* [34], thus, phages that infect *Gordonia sp*. are of interest because they may also have host range to *M*. *tuberculosis* and other important pathogenic bacteria.

The results show that there is a high level of diversity of the putative lysis genes within this set of 77 phages that results in 16 different genome organizations, many of which are distinct from the lysis cassettes that have been previously evaluated. The most diverse set of proteins identified in the lysis cassettes are the transmembrane holin-like proteins. In the current study, 34 different genes encoding holin-like proteins were identified as well as a potential holin-major capsid fusion protein. The holin-like proteins contained between 1–4 TM helices, the genes are not shared to a high degree amongst the 16 reference phages and are present in the lysis cassette in a wide range of combinations of up to 4 genes in which none are duplicated. Detailed evaluation of the TM domains and predicted membrane topologies of the encoded holin-like proteins show that many have novel organizations that have not been previously described. These findings suggest that the organization and diversity of genes within the lysis cassette may be important in phage fitness and provide compelling support that there are novel operational lysis models yet to be discovered.

## Methods

### Identification of reference phages utilized in this study

The Actinobacteriophage Database at PhagesDB [40] contains all of the genomic information for phages that have been collected through the SEA-PHAGES consortium [41]. Individual genes from phages within the PhagesDB are grouped into distinct Phamilies (Phams based on clustering the protein sequences with MMseqS2 [42] utilizing the following commands:

1^st^ iteration: mmseqs cluster–min-seq-id 0.35 -c 0.8 -e 0.001 -s 7 –cluster-steps 1
2^nd^ iteration: mmseqs search–min-seq-id 0.15 -c 0.7 –cov 0.7 -e 0.001 –e-profile 0.001 -s 7 –num-iterations 3

Comprehensive analysis on the full dataset has shown that these thresholds assemble phams with significantly similar sequences that appear to be functionally related (C. Gauthier, Univ. Pitt. manuscript in preparation). Phages that infect *Gordonia* strains are then clustered based on the percentage of genes that they have in common [25]. In June 2022, there were 81 phages listed in PhagesDB that infect *Gordonia rupripertincta* NRRL B-16540 of which 54 had GenBank entries. The 81 phages were found in 13 clusters (CP, CS, CT, CV, CW, CZ, DG, DJ, DM, DQ, DR, DV, DZ) and also included the singleton phage VanLee. All phages within a single cluster were visualized by utilizing Phamerator with default settings [43] to evaluate the genome alignments and identify the predicted lysis cassette region by identifying the location of the annotated endolysin (lysin A) gene/s. Once this region was identified, the five genes directly up and downstream of the endolysin were evaluated to determine if they were assigned to the same Pham. Individual phages *within each cluster* were then grouped based on an analysis of the pham membership and order of the five genes flanking the putative endolysin; exact matches were required for sorting into discrete lysis cassette groups. This allowed the creation of different groups of phages within each cluster that had distinct genome organizations at the lysis cassette. This approach revealed three distinct groups within cluster CT and two within clusters DV and DQ. All other clusters either showed the same exact lysis cassette gene organization or only had a single phage within the cluster. Clusters CW and DZ did not have phages that had been annotated at the time of this analysis, therefore these clusters were omitted from this analysis. Thus, the analysis resulted in the finding of 16 different genome organizations at the putative lysis cassette that encompassed a total of 77 phages. A reference phage was then selected for each of the 16 genome organizations each to represent the similar phages within each group. Reference phages were selected based on having a complete annotation in GenBank and being isolated/annotated by students at USF or Chatham. This accounted for six of the 16 reference phages. The others were selected randomly from each group or by virtue of being the only phage in the cluster (cluster CV and DG1). The reference phage for each group is shown in Table 1.

**Table 1:** *Gordonia* phages highlighted in this study.

	Phage	Cluster	Phages in Cluster	Accession #	Life Cycle	Morphotype	Genome Size (bp)	GC content (%)
*1*	Clawz	CP	2	MT498058	unknown	Siphoviridae	57664	64.9
*2*	PokyPuppy	CS	4	ON456331	lytic	Siphoviridae	77065	59.1
*3*	Amok	CT	11	OL829977	lytic	Siphoviridae	45278	61.1
*4*	Mayweather	CT	8	MN062716	lytic	Siphoviridae	48382	60.6
*5*	Orla	CT	4	MT889367	lytic	Siphoviridae	47354	63.3
*6*	KappaFarmDelta	CV	1	ON970567	temperate	Siphoviridae	49606	67.3
*7*	Suerte	CZ	4	MN428057	temperate	Siphoviridae	47306	66.5
*8*	LilyPad	DG	1	ON970620	lytic	Siphoviridae	64158	59.2
*9*	Secretariat	DJ	16	MT310850	lytic	Siphoviridae	57731	52.8
*10*	EpicDab	DM	1	MK660712	temperate	Siphoviridae	16658	69.1
*11*	ChisanaKitsune	DQ	3	MZ820089	lytic	Myoviridae	88657	60
*12*	DalanDe	DQ	1	ON755184	lytic	Siphoviridae	85762	60.2
*13*	CaiB	DR	5	ON108644	lytic	Siphoviridae	61620	68.6
*14*	DumpTruck	DV	1	MZ005671	unknown	Siphoviridae	67754	57.9
*15*	Jalebi	DV	14	OL455895	unknown	Siphoviridae	68501	58.4
*16*	VanLee	Singleton	1	MZ028627	temperate	Siphoviridae	84560	67.8

### Bioinformatic analyses and characterization of endolysins

The endolysin genes, lysin A and lysin B, were first identified through analysis of the phage genomes in GenBank, Phamerator and the Actinobacteriophage Database at PhagesDB as detailed above. The LysA and LysB protein sequences were then collected for all reference phages and organized based on their Pham membership. All query proteins were analyzed by HHpred [44] to identify predicted functional domains. Default settings were used in HHpred to search the PDB_mmCIF70_14_Apr, Pfam-A_v35 and NCBI_Conserved_Doains(CD)_v3.19 databases. The cutoff used for considering HHpred hits was >95% probability and e values of at least 1 x 10^−20^. Any hits at or above these values were evaluated to determine the consensus of the functional hits with emphasis of observing consistency in function calls between the PDB, conserved domain (cd) and protein family (pfam) hits. This information was utilized to assign functional domains to the full query sequence. Once all domains were assigned, LysA and LysB proteins were grouped with respect to their conserved domains and predicted activities.

### Bioinformatic analyses and characterization of transmembrane proteins

The number and location of predicted transmembrane helices (TM) were identified within query protein sequences utilizing three different programs: SOSUI [45], Deep TMHMM [46], and TOPCONS [47]. SOSUI and Deep TMHMM predict TMs using a single analysis, while TOPCONS provides a consensus predication based on five different analysis within the program. Query sequences were also evaluated with SignalP-5.0 [48] and LipoP [49] to evaluate the presence of a signal sequence and to determine if the TM region might serve as a lipoprotein anchor sequence. Note that TOPCONS and Deep TMHMM also predict signal sequences. Finally, all query proteins were evaluated in HHpred as described for the endolysins to determine if transmembrane domains or signal sequences were predicted. The data for each query protein from each analysis program was collected in a spreadsheet and coded for: 1), TMs identified by HHpred (yes/no), 2) predicted number of TM helices by SOSUI, Deep TMHMM and TOPCONS (0–4), 3) predicted protein topology in the membrane (ex. N-in; C-in), 4) location of each of the helices by amino acid number (ex: 22–44), 5) distance of the first and last helix from the N and C-terminus to establish how much of the protein would be found in or out of the cell (ex: N-term = 10 amino acids inside cell) and 6) prediction of a signal sequence by any of the programs (signal/no). The final number of TM domains was determined by the consensus prediction of SOSUI, Deep TMHMM and TOPCONS. A complied dataset and notes regarding the analysis of each protein is shown in S1 Table. For the 46 proteins evaluated in this study, there was 100% consensus for the number and location of the predicted TM domains for 38 of the proteins (81%). Six additional proteins had TM domains identified by the majority of programs, with one of the programs differing only in the number of TM domains predicted and thus, the consensus was used. For the remaining three sequences, the three programs differed in predicting the nature of the helices as being either a TM or a signal peptide. In these instances, the consensus prediction of a signal sequence was based on having four of the five programs predict a signal. The amino acid composition and organization of specific TM domains was evaluated using wheel projections of the helix from SOSUI. Analysis of coiled-coil regions was performed using Waggawagga [50], Multicoil [51], Prabi-Gerland Coil-Coil Prediction [52] and HHpred all with default parameters. All endolysin proteins that were identified in this study were also evaluated with SOSUI, Deep TMHMM, TOPCONS and SignalP to identify possible SAR-like sequences.

## Results and discussion

### Characterization and organization of endolysins (Lysin A) in phages that infect *G*. *rubripertincta*

For effective cell lysis, phages that infect Gram-positive bacteria require an endolysin enzyme (also termed lysin A or LysA) for digestion of the peptidoglycan (PG) layer of the cell wall and deletion of the LysA results in a non-lysis phenotype [28, 30, 53]. These enzymes have received more attention than others in the lysis cassette due to their possible use in antibacterial applications [54–57]. In this regard, the number of published crystal structures to enzymes that target the various PG bonds has greatly facilitated the ability to bioinformatically identify and characterize the endolysin genes from diverse phages. In phages that infect Gram-positive bacteria, the majority of phage endolysins that have been bioinformatically and functionally evaluated are from mycobacteriophages [22–25]. The current data show that the lysA genes from mycobacteriophages are highly mosaic and typically encode a single LysA protein that has multiple enzymatic domains. These enzymes are highly diverse at the amino acid level yet share a common modular structure in which an N-terminal domain and one or more central domains harbor activities that target distinct bonds within the PG. In addition, most enzymes also contain a C-terminal PG/cell wall binding domain.

To identify the location of the annotated endolysin or *lysA* genes in the 16 reference *Gordonia* phages, each genome was manually reviewed in Phamerator and Genbank as detailed in Methods. Table 2 shows the location of the predicted endolysin or *lysA* genes from each of the reference phages and they are sorted by their Pham designations. Every *Gordonia* phage evaluated had at least one annotated endolysin or *lysA* gene with Amok, CaiB, EpicDab, KappaFarmDelta, Orla, PokyPuppy and Mayweather having two distinct annotated *lysA* genes. Overall, there were 23 *lysA* genes identified within the 16 phages that grouped into 10 different Phams. To characterize the predicated domains and activities, the LysA enzymes were evaluated using HHpred as described in Methods. Five enzymatic domains were identified across the 23 proteins based on high coverage to the query, low e value scores and consistency in the activities across the PDB, conserved domain, CATH and pfam hits. Table 3 shows the summary of the PDB, cd, CATH and pfam hits and the predicted function for the various domain hits. For consistency, the terms Ami-2A, M15, M23, GH43 and GH25 were used for the domains to reflect similar domains identified in mycobacteriophage LysA [see Table 1 in ref. 22]. The Ami-2A domain was most identified across the LysA proteins and this domain had high coverage hits to PDB 3D2Y_A N-acetylmuramoyl-L-alanine amidase from *E*.*coli* [58] and PDB 6SSC_A N-acetylmuramoyl-L-alanine amidase from *C*. *intestinale* [59]. This domain also had cd hits to 06583 (peptidoglycan recognition proteins) and pfam hits to 01510.28 (Amidase family). These findings are consistent with the work in mycobacteriophages that also found the Ami-2 domain to be the most prominent [22]. In addition, domains were identified in the *Gordonia* phage LysA proteins that folded with high probability and low e values to several PDBs with predicted C-terminal cell wall binding domains (CW1 and CW2) [60, 61] or had less defined enzymatic activity (GS25?). However, unlike the mycobacteriophages where the C-terminal cell wall binding domains were identified in a high number of LysA proteins, only 6 of the 16 *Gordonia* phages had defined CW binding regions through HHpred analysis.

**Table 2:** Identification of *lysA* genes.

	Reference Phage	*lysA* gene	cluster	Pham	Pham designation used for schematics	SAR domain identified
*1*	**KappaFarmDelta**	**gp20**	**CV**	**602**	**A**	NO
*2*	**PokyPuppy**	**gp33**	**CS**	**1295**	**B**	NO
*3*	**PokyPuppy**	**gp34**	**CS**	**1448**	**C**	NO
*4*	Clawz	gp65	CP	5603	D	NO
*5*	DalanDe	gp55	DQ	5603	D	NO
*6*	**ChisanaKitsune**	**gp56**	**DQ**	**5603**	**D**	NO
*7*	**Secretariat**	**gp9**	**DJ**	**7090**	**E**	NO
*8*	**EpicDab**	**gp5**	**DM**	**11767**	**F**	NO
*9*	**EpicDab**	**gp6**	**DM**	**12044**	**G**	NO
*10*	Amok	gp19	CT	27556	H	NO
*11*	Mayweather	gp21	CT	27556	H	NO
*12*	Orla	gp22	CT	27556	H	NO
*13*	**CaiB**	**gp43**	**DR**	**27556**	**H**	NO
*14*	Suerte	gp19	CZ	31868	I	NO
*15*	LilyPad	gp31	DG	31868	I	NO
*16*	DumpTruck	gp32	DV	31868	I	NO
*17*	Jalebi	gp35	DV	31868	I	NO
*18*	**VanLee**	**gp28**	**Singleton**	**31868**	**I**	NO
*19*	**CaiB**	**gp44**	**DR**	**31879**	**J**	NO
*20*	Amok	gp20	CT	31879	J	NO
*21*	Mayweather	gp22	CT	31879	J	NO
*22*	Orla	gp23	CT	31879	J	NO
*23*	KappaFarmDelta	gp22	CV	31879	J	NO

Phages are sorted based on the designated Pham number from PhageDB.org as of 6.15.2022. Several phages have multiple *lysA* genes and are listed twice (note the difference in the gene number). Each Pham is designated with a letter (A-J) and this designation is used in the lysis cassette scenarios for ease of comparing the *lysA* in different gene organizations. All of the proteins were evaluated for TM domains that might serve as a SAR as detailed in Methods. Bolded phages serve as representatives for the Pham in later figures.

**Table 3:** Domain features of bioinformatically-defined *Gordonia* phage endolysin (LysA) domains.

Domain	Putative Activity	Ref PDB hit	PDB reference	Organism	cd Hit	PFAM hit	CATH	Reference *Gordonia* Phage
M23	M23 peptidase	2HSI_B	63	*P*. *aeruginosa PAO1*	cd12797	PF01551.25	2.70.70.10	PokyPuppy gp34
	M23 peptidase	6JMY_A	64	*Campylobacter jejuni*				
GH43	Glycoside hydrolase	4HBS_A	62	*Bacteroides ouatus*	cd08772			ChisanaKitsune gp56
GH25	Glycoside hydrolase	2WAG_A	65	*Bacillus anthracis*	cd06417	PF01183.23		CaiB gp44
M15	L-Ala-D-Glu peptidase (M15-like)	2MXZ_A	66	Enterobacteria phage T5	cd14814	PF13539.9		CaiB gp43
Ami-2A	N-acetylmuramoyl-L-alanine amidase	3D2Y_A	58	*E*. *coli*	cd06583	PF01510.28	3.40.80.10	Secretariat gp9
	N-acetylmuramoyl-L-alanine amidase	6SSC_A	59	*C*. *intestinale*				
GH25?	putative hydrolase (GH25 family)				cd06418			ChisanaKitsune gp56
CW1	Cell wall binding? LGFP repeat-containing protein	6SX4_CCC	60	*C*. *glutamicum*				VanLee gp28
CW2	PGBD-like superfamily	4BXJ_A	61	*P*. *aeruginosa PAO1*			1.10.101.10	Secretariat gp9

**Fig 2:**
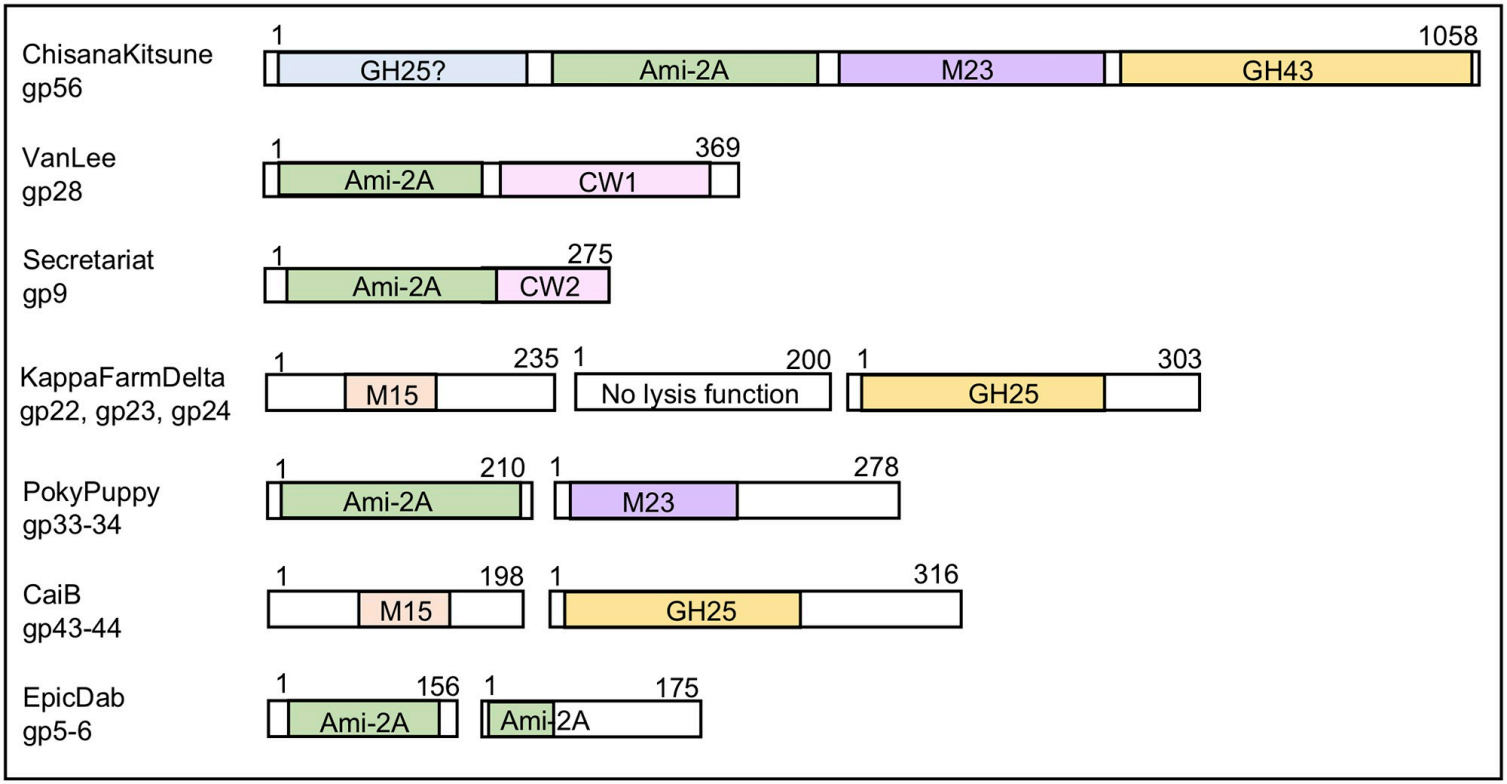
Organizations and modular structure of *lysA* genes from *Gordonia* phages. Based on the domains identified in each LysA (Table 3), 7 different organizations are observed in the 16 reference phages. The colored area of each LysA represents a different enzymatic domain and the size of the shading shows the coverage within each LysA. The specific activities associated with each domain are described in Table 3. Note that gp23 in KappaFarmDelta that has no evidence for lysis activity but is shown because it is located between both of the defined *lysA* genes (gp22 and gp24).

All LysA proteins were evaluated in HHpred as detailed in Methods. Conserved domains are shown for representative phage proteins with PDB, cd, pfam and CATH hits. The Domain designations (column 1) are used in the schematics presented in Fig 2.

Based on the predicted activities assigned to each of the reference phage LysA proteins and the genomic organization of the genes, there were 7 different domain combinations across the 16 *Gordonia* phages. A single phage was selected to represent each of the combinations and the schematics are presented in Fig 2. Nine of *Gordonia* phages express a single LysA that is predicted to have multiple enzymatic domains. However, unlike the three-domain structure typically identified in the majority of the LysA proteins from mycobacteriophages, only Claws, DalanDe and ChistanaKitsune encode a LysA with more than two domains. The LysA from these phages is noteworthy as the protein is nearly 1,100 amino acids making it twice as large as any LysA enzymes described in mycobacteriophages. These LysA proteins have four predicted domains including an N-acetylmuramoyl-L-alanine amidase (Ami-2A), a glycoside hydrolase (GH43 family) [62], a M23 peptidase (M23) [63, 64], and an N-terminal region that may also have additional glycoside activity (GS25?) due to a hit to cd06418 (GS25 family) [65]. Five other phages encode an ~350 amino acid LysA with two domains that is represented by gp30 from VanLee. This LysA has a N-terminal domain predicted with Ami-2A activity and a C-terminal region with a predicted cell wall binding function (CW1) that hits to LGFP repeat-containing proteins. Finally, Secretariat (gp9) has a smaller LysA (275 amino acids) with predicted N-terminal Ami-2A activity and a different cell all binding domain (CW2). It is noteworthy that the LysA from Secretariat hits to the hydrolase from *P*. *aeruginosa* PAO1 (PDB 4BXJ_A) [61] with coverage across the entire query including the C-terminal 80 amino acids of Secretariat that the other subjects do not cover. This C-terminal region of the putative hydrolase contains a predicted PG binding domain (CW2). The last seven of the reference phages have two distinct *lysA* genes and each encodes an enzyme with a single predicted enzymatic domain. There are four different combinations of the activities across these seven phages and these are shown in Fig 2 and discussed below.

All of the *Gordonia* phages evaluated have a l*ysA* that can be validated bioinformatically with high probability matches to relevant PDB structures that have activities toward the PG. When all of the specific enzymatic activities for each LysA are considered in the context of the 7 different genome organizations shown in Fig 2, there are essentially five predicted enzymatic scenarios present across the 16 phages evaluated. First, six phages (Secretariat, VanLee, Jalebi, DumpTruck, LilyPad, and Suerte) have a single LysA enzyme that contains a single Ami-2A activity toward the MurNac—L-Ala linkages between the glycan and peptide where all of these domains fold on the same PDB entries and hit the same conserved domains and pfams. These phage also have a predicted C-terminal cell wall binding domain. Second, ChisanaKitsune, Clawz and DalanDe have an exceptionally large LysA with four predicted activities toward: 1) the MurNac—L-Ala linkages, 2) the glycan linkages, 3) the peptide bonds within the PG and 4) a possible second activity toward the linkages in the glycan. Third, five phages (CaiB, Mayweather, Amok, Orla and KappaFarmDelta) are predicted to express two LysA proteins each with a distinct activities; one toward the cleavage of the β -(1,4) linkages between the MurNAc and GlcNAc in the glycan but also an activity that targets the peptide bonds within the PG (M15 peptidase domain) [66]. Fourth, PokpPuppy also expresses two LysA proteins but with activities targeted to the MurNac—L-Ala linkages between the glycan and peptide and the other toward the peptide bonds within the PG (M23 peptidase domain). Finally, EpicDab expresses two LysA enzymes, but both are much smaller than those from the other *Gordonia* phages (<175 amino acids) and both appear to have only activity targeted to the MurNac—L-Ala linkages between the glycan and peptide.

### Detection and characterization of Lysin B in phages that infect *G*. *rubripertincta*

*Gordonia* strains have a cell wall that belongs to wall chemotype IV sensu Lechevalier and Lechevalier and contains an outer membrane consisting of 44–66 carbon mycolic acids that are covalently attached to the arabinogalactan-peptidoglycan complex [19–21]. This represents a barrier to lysis that the LysA enzymes do not have specificity to target. Thus, it has been demonstrated that many phages that infect bacterial strains with a complex mycomembrane express a second endolysin protein termed lysin B (LysB). LysB targets the linkage between the mycolic acids and the arabinogalactan-peptidoglycan layer to separate the OM from the PG [27–30]. This is proposed to represent the third component of the lysis pathway in Gram-positive bacteria and is correlated to the spanin function in Gram-negative bacteria [6, 7, 23].

To identify the *lysB* in each of the 16 reference *Gordonia* phage genomes, each was manually reviewed in GenBank and Phamerator to identify the location of the putative *lysB*. Table 4 shows the location of the annotated *lysB* genes from each of the reference phages and their Pham designation. Every *Gordonia* phage evaluated had an annotated *lysB* gene except Claws (cluster CP). The LysB proteins ranged from 247–287 amino acids and unlike the LysA proteins all but the LysB from EpicDab and PokyPuppy was grouped to the same Pham. To validate the *lysB* annotation, each protein was evaluated in HHpred as detailed in Methods. Every one of the predicted LysB proteins modeled with near full coverage to the crystal structure 3HC7_A from the LysB of mycobacteriophage D29 [28]. The hit to PDB 3HC7_A was the top hit for every LysB sequence, showed >99% probability, >95% query coverage and e scores < 10^−21^. All proteins also had numerous other high probability hits to acetyl xylan esterases and cutinases and hit to many of the same PDB hits as the LysB from D29 (S2 Table). LysB has a conserved GxSxG motif found in lipolytic enzymes [27, 67], and the 3D structure of D29 LysB identified Ser82, Asp166, and His240 as the residues at the active site, where S82 is central in the GxSxG motif [28]. The analysis of the *Gordonia* phages showed that 12 of the LysB proteins have the conserved GxSxG motif, while VanLee and LilyPad have an AxSxG motif and DalanDe has a GxSxh, thus, all have the conserved D29 Ser82 equivalent (Table 4). In addition, analysis of the HHpred alignments showed that the *Gordonia* LysB proteins had conserved aspartic acid and histidines that aligned with Asp166 and His240 of D29 LysB. These results provide compelling evidence for the identification of the *lysB* gene in 15 of 16 reference phages that infect *G*. *rubripertincta*.

**Table 4:** Identification and characterization of *lysB* genes and proteins.

Reference Phage	Cluster	lysB Gene	LysB size in amino acids	Pham	GxSxG motif	D29 LysB D166 match	D29 LysB H240 match	Top HHpred PDB hit	E-value	lysB Genome Location
PokyPuppy	CS	gp49	283	41076	GySlG	YES	YES	3HC7_A	2.70E-28	distal
Amok	CT	gp46	279	40691	GySqG	YES	YES	3HC7_A	8.80E-25	distal
Mayweather	CT	gp51	265	40691	GySlG	YES	YES	3HC7_A	2.30E-23	distal
Orla	CT	gp53	283	40691	GySlG	YES	YES	3HC7_A	4.70E-27	distal
KappaFarmDelta	CV	gp29	262	40691	GySaG	YES	YES	3HC7_A	2.50E-25	distal
LilyPad	DG	gp90	237	40691	AySlG	YES	YES	3HC7_A	2.30E-23	distal
Secretariat	DJ	gp28	262	40691	GySgG	YES	YES	3HC7_A	1.60E-25	distal
DalanDe	DQ	gp26	231	40691	GySlh	YES	YES	3HC7_A	6.20E-22	distal
ChistanaKitsune	DQ	gp31	238	40691	GfSlG	YES	YES	3HC7_A	2.90E-21	distal
DumpTruck	DV	gp89	287	40691	GfSaG	YES	YES	3HC7_A	1.10E-23	distal
VanLee	Singleton	gp104	244	40691	AySlG	YES	YES	3HC7_A	3.10E-24	distal
Suerte	CZ	gp23	262	40691	GySaG	YES	YES	3HC7_A	3.60E-27	LC
EpicDab	DM	gp7	284	41054	GySqG	YES	YES	3HC7_A	4.30E-27	LC
CaiB	DR	gp45	240	40691	GySqG	YES	YES	3HC7_A	3.10E-23	LC
Jalebi	DV	gp86	283	40691	GySgG	YES	YES	3HC7_A	1.70E-22	LC

Pham number is from PhageDB.org as of 6.15.2022. Location: distal = *lysB* gene found in distal portion of genome >4,500bp from *lysA*. LC = *lysB* found within a putative lysis cassette with *lysA*.

### Identification and characterization of transmembrane proteins

In λ, phage 21 and phage P2, the *lysA* is typically associated within a defined linear lysis cassette region of the phage genome that also contains the holins and other genes involved in the lysis pathway [Fig 1 and ref: 6, 7]. In the mycobacteriophages, a similar organization has been observed for the majority of phages with many also containing the *lysB* gene [22, 23]. In contrast, analysis of 60 *Gordonia* phages showed that the predicted *lysB* was often found at a distal location to the *lysA* and putative holins [25]. Thus, the location of the *lysA* can best inform the bioinformatic identification of putative holins and also the identification of other proteins that might be involved in the lysis pathway. Since the *lysA* was identified in all of the 16 reference phages, the protein products from the genes that were within 10 genes either up or downstream of the *lysA* were evaluated for TM helices as described in Methods. A similar analysis was applied to the genes up and downstream of the *lysB* if the gene was localized to a region of the genome distal to the *lysA*.

The results show that there are 46 proteins with one to four predicted TM helices that are present in a defined lysis cassette of the 16 reference phages (Table 5). The detailed results of the analysis are presented in S1 Table. All proteins with two or more TM domains are predicted as membrane proteins by all programs. In addition, all 46 proteins are predicted to have TM helices by HHpred and 29 of the 46 have modest hits to holin pfams (S1 Table). All but four of the TM proteins (gp 16–19 from CaiB, discussed in detail below), are found directly downstream of the *lysA* genes, with no TM proteins identified adjacent to any of the distal *lysB* genes. Of the 46 proteins identified, one is predicted to have a signal sequence and to be secreted (Amok gp23). One protein is predicted to have a signal sequence and also an internal TM helix (DumpTruck gp33). Ten proteins are predicted to have a single TM helix, 15 are predicted to have two TM helices, one is predicted to have three TM helices and 18 are predicted to have four TM helices. Importantly, the 46 TM proteins group to 34 distinct Phams meaning and only nine of the genes are shared amongst the reference phage genomes. Only gp58 and gp60 from the DQ phage DalanDe appear unique as these are orphams and have no additional Pham members. However, the other 32 Phams contain between 5 to 200 members, 17 of these have members from phages that are grouped to at least two other clusters and infect strains other than *G*. *rubripertincta* (Table 5). Thus, for this report, the proteins will be referenced as “holin-like” since they all have TM helices and all are found in a cassette with a validated endolysin.

**Table 5:** TM proteins Identified in 16 reference phages.

	Phage	Gene	Size (amino acids)	TM helices	Cluster	Pham	Number of Pham members	Pham designation used for schematics	Clusters with Pham Members
*1*	CaiB	gp18	153	4	DR	309	205	**1**	B, CS, DR, W, CZ, DG, DS, DR, AD, DK, DB, DO
*2*	DumpTruck	gp35	162	4	DV	309	205	**1**	
*3*	LilyPad	gp34	162	4	DG	309	205	**1**	
*4*	PokyPuppy	gp36	180	4	CS	309	205	**1**	
*5*	KappaFarmDelta	gp23	150	4	CV	582	142	**2**	DE, DC, CV, CY, DB, CZ, DW, DT
*6*	Amok	gp21	93	2	CT	697	136	**3**	CT, CZ, CA, CR, DI
*7*	CaiB	gp19	103	1	DR	824	114	**4**	B, DR, W, AD, DO
*8*	PokyPuppy	gp37	110	1	CS	1647	53	**5**	CS
*9*	PokyPuppy	gp38	79	1	CS	1730	49	**6**	CS
*10*	Amok	gp22	162	4	CT	1770	48	**7**	CT, O
*11*	Jalebi	gp37	90	1	DV	2393	35	**8**	CZ, DV
*12*	Suerte	gp22	168	2	CZ	2746	29	**9**	CZ, DE, DA, CD
*13*	Secretariat	gp10	147	2	DJ	2806	28	**10**	DJ
*14*	Amok	gp23	102	Signal	CT	2974	27	**11**	CT
*15*	DumpTruck	gp33	87	Signal + 1	DV	3648	20	**12**	DV, DG, CD
*16*	Jalebi	gp36	89	2	DV	3648	20	**12**	
*17*	Suerte	gp20	126	2	CZ	3865	19	**13**	CZ
*18*	Mayweather	gp25	187	2	CT	5203	11	**14**	CT, DX
*19*	DumpTruck	gp36	131	1	DV	5805	9	**15**	DG, DV
*20*	LilyPad	gp35	122	1	DG	5805	9	**15**	
*21*	ChisanaKitsune	gp57	179	2	DQ	7032	7	**16**	DQ, DX
*22*	DalanDe	gp56	172	2	DQ	7032	7	**16**	
*23*	Orla	gp24	111	2	CT	7643	6	**17**	CT
*24*	ChisanaKitsune	gp59	164	4	DQ	8802	5	**18**	DQ
*25*	DalanDe	gp59	163	4	DQ	8802	5	**18**	
*26*	ChisanaKitsune	gp60	112	1	DQ	10884	3	**19**	DQ
*27*	Clawz	gp66	135	2	CP	12851	2	**20**	CP
*28*	VanLee	gp30	161	4	sing	27436	211	**21**	A, K, CZ, H, DT, DP
*29*	Orla	gp26	148	2	CT	32035	65	**22**	DE, CZ, CV, CY, CP, DB, DW, DH, DN
*30*	KappaFarmDelta	gp24	107	1	CV	32537	6	**23**	CV, DC
*31*	DalanDe	gp60	104	1	DQ	32846	1	**24**	DQ
*32*	DalanDe	gp58	101	2	DQ	32860	1	**25**	DQ
*33*	Jalebi	gp39	128	4	DV	33145	116	**26**	Z, CZ, CY, CQ, DC, F, DB, AB, DV, DN
*34*	KappaFarmDelta	gp26	116	3	CV	33145	116	**26**	
*35*	VanLee	gp31	145	1	sing	33261	65	**27**	DE, CZ, DT, DP, DA
*36*	LilyPad	gp32	134	4	DG	33305	50	**28**	BH, DG, CT, DS, BM, DA, DB, DS
*37*	Mayweather	gp23	116	4	CT	33305	50	**28**	
*38*	CaiB	gp17	164	4	DR	38577	112	**29**	B, DR, W, AD, DG
*39*	DumpTruck	gp34	150	4	DV	38611	92	**30**	CS, CZ, DG, DB, DK, DO
*40*	LilyPad	gp33	148	4	DG	38611	92	**30**	
*41*	PokyPuppy	gp35	148	4	CS	38611	92	**30**	
*42*	CaiB	gp16	133	4	DR	38627	84	**31**	B, DR, AD, DD
*43*	VanLee	gp29	113	4	sing	38803	39	**32**	DN, CZ, CY, DW
*44*	Mayweather	gp24	78	2	CT	38944	20	**33**	CT, CP, DX
*45*	Orla	gp25	72	2	CT	38944	20	**33**	
*46*	Suerte	gp21	77	2	CZ	38945	20	**34**	DA, CZ, CD

Proteins are sorted by Pham number from PhageDB.org as of 6.15.2022. Proteins that are grouped within the same Pham are shaded. The numbers of TM helices reflect the consensus call and detailed data for each protein can be found in S1 Table. Each Pham is designated with a number (1–34) and this designation is used in the lysis cassette schematics for ease of comparing the proteins within the different gene organizations.

#### Topologies and characterization of proteins with one TM helix

The Type III holins are the simplest proteins containing a single TM helix and are typified by phage T4 protein T that is 218 amino acids and has a predicted N-in-C-out topology with a168 amino acid extracellular C-terminal domain (Table 6 and [68, 69]). Protein T has canonical holin function and is capable of oligomerizing into large holes that allow exit of the endolysin [68, 69]. In the current report there are 11 proteins with one predicted TM helix that are grouped into 10 different Phams and also gp23 from Amok that is predicted to be secreted via a N-terminal TM helix that serves as a signal sequence (Table 6). The 10 proteins predicted to be inserted in the membrane show a distinct structure and topology to the gene T Type III holin. First, the proteins are much smaller than protein T and range from 79–145 amino acids (Table 6). Second they all have a predicted N-out-C-in topology with a very small N-terminal domain outside the cell and a cytoplasmic C-terminal domain of between 52–115 amino acids that encompasses the bulk of the protein (Table 6). Third, analysis of the amino acid composition of the entire C-terminal cytoplasmic domains shows that there are no predicted coiled-coil structural motifs. Finally, the cytoplasmic domains not only vary in length but also vary in the number of charged RKHDE residues. Six of the ten proteins show >30% charged residues with VanLee gp31 and LilyPad gp35 showing a remarkable 46% RKHDE content. Interestingly, the proteins with one TM helix are always found in a linear array with at least two additional proteins with that usually have two or more TM helices (see the schematics and Discussion in later sections).

**Table 6:** Characterization of proteins with 1 TM helix.

	Phage	Gene	Cluster	Pham designation from Table 5	Size of protein (amino acids)	N-terminal amino acids Outside Cell	C-terminal amino acids Inside Cell	Total C-terminal RKHDE resides	Percent RKHDE residues in C-terminal domain
*1*	Jalebi	gp37	DV	**8**	90	**17**	**52**	**13**	**25%**
*2*	CaiB	gp19	DR	**4**	103	**4**	**77**	**20**	**26%**
*3*	PokyPuppy	gp37	CS	**5**	110	**4**	**87**	**26**	**30%**
*4*	PokyPuppy	gp38	CS	**6**	79	**4**	**54**	**20**	**37%**
*5*	DumpTruck	gp36	DV	**15**	131	**4**	**106**	**36**	**34%**
*6*	LilyPad	gp35	DG	**15**	122	**4**	**96**	**44**	**46%**
*7*	ChisanaKitsune	gp60	DQ	**19**	112	**5**	**85**	**31**	**36%**
*8*	KappaFarmDelta	gp24	CV	**23**	107	**8**	**76**	**20**	**26%**
*9*	DalanDe	gp60	DQ	**24**	104	**5**	**79**	**27**	**34%**
*10*	VanLee	gp31	singelton	**27**	145	**11**	**115**	**53**	**46%**
*11*	Amok	gp23	CT	**11**	102	**secreted**			
	**Phage**	**Gene**	**Cluster**	**Pham designation from** Table 5	**Size of protein (amino acids)**	**N-terminal amino acids Inside Cell**	**C-terminal amino acids Ouside Cell**	**Total N-terminal RKHDE resides**	**Percent RKHDE residues in C-terminal domain**
	T4	gene *t*	N/A	N/A	218	**29**	**167**	**6**	**4%**

Proteins that are grouped to the same Phams are shaded. Information on protein T from phage T4 is provided in the last row for comparison. N/A = not applicable.

#### Topologies and characterization of proteins with two TM helices

**Fig 3:**
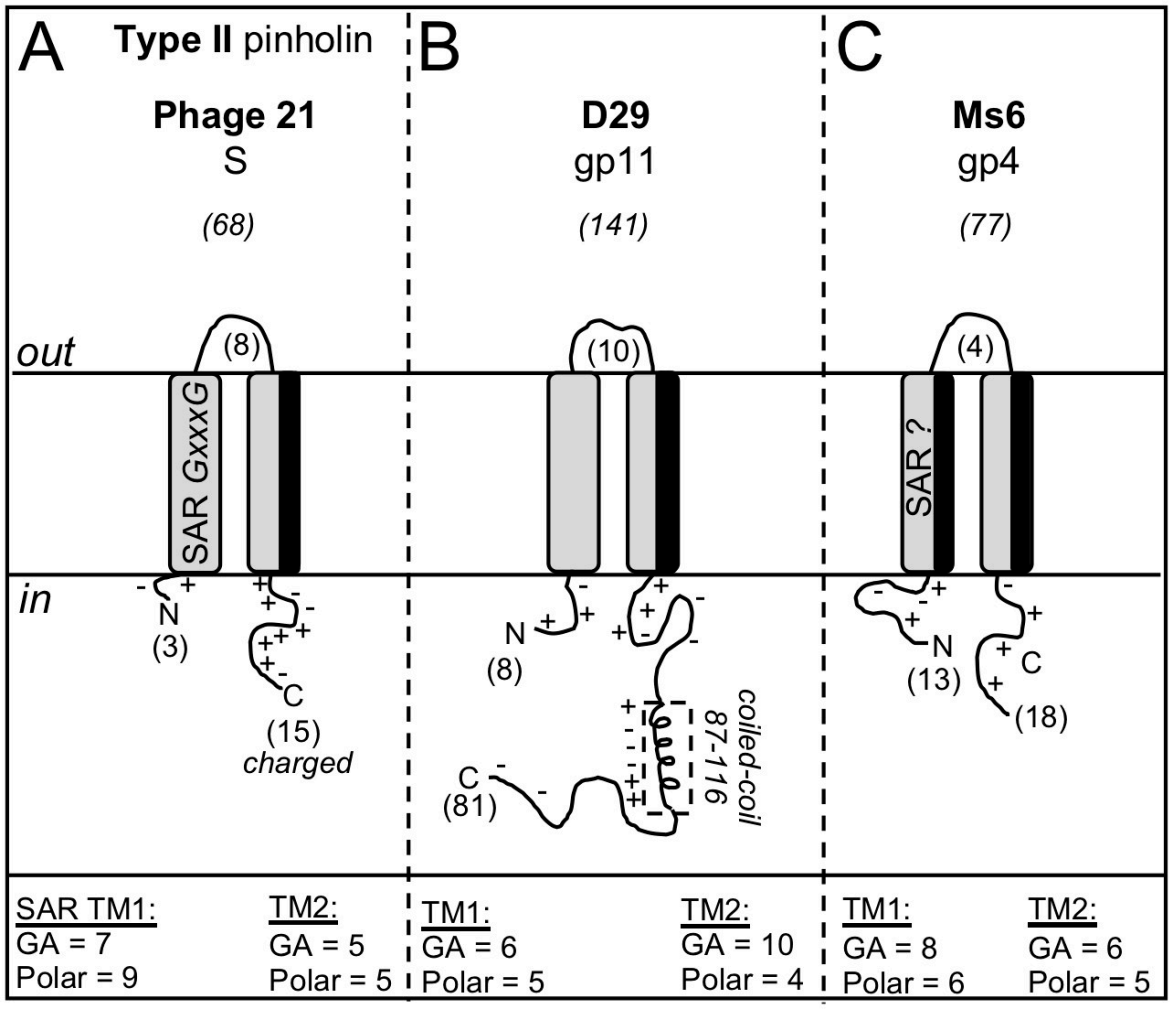
Validated holins with 2 TM helices. TM helices are indicated by the gray areas spanning the membrane. TM helices with dark shading to one side indicate a hydrophilic face. Coiled-coil regions are shown as helix within a dashed box. Amino acid characteristics of TM1 and TM2 are shown in the bottom panel: GA = number of glycine and alanine residues in the helix; Polar = number of polar residues in the helix (STYQNDEKHR). N = N-terminal; C = C-terminal;+ = location of a positive residue (RKH);— = location of a negative residue (DE); numbers in parenthesis indicate the number of amino acids in a given domain or the size of the reference protein above the phage name. SAR = Signal arrest release sequence. GxxxG = the presence of a GxxxG region in the TM helix.

The three most studied holin proteins with two TM helices include the Type II pinholins that are typified by R^21^68 of phage 21 [6, 7], gp4 from mycobacteriophage Ms6 [23, 31–33] and gp11 from mycobacteriophage D29 [31, 70–73]. Each of these holins has a distinct topology and these are shown in Fig 3. The Type II pinholins do not create large concatemers that allow release of macromolecules through the membrane, but function to destabilize the PMF and control the timing of the lysis cycle. In addition to the two TM domains, R^21^68 has several structural features that have been determined to be important in the function as a pinholin. These include, 1) a N-in-C-in topology, 2) a TM1 helix that has a high glycine and alanine (GA) content and also a high percentage of hydrophilic residues (>30%) that allow the domain to function as a SAR and exit the membrane following loss of the PMF [17, 18], 3) a TM2 helix with asymmetric polar residues and GxxxG regions predicted to assist in the homotypic interactions of the TM2 [17, 18] and 4) a short C-terminal domain that is composed of >30% charged (RKHDE) residues (Fig 3, panel A). The gp11 holin protein of mycobacteriophage D29 also contains two TM domains with a N-in-C-in topology but is much larger than R^21^68 and has an extended C-terminal domain of 81 amino acids (Fig 3, panel B). In contrast to the function of R^21^68 as a pinholin, gp11 is capable of oligomerizing and forming large holes through the TM1 helix [31, 72]. Neither of the D29 gp11 TM helices have GxxxG motifs or a high proportion of hydrophilic residues although the TM1 helix is proposed to covert from an α-helix to β-sheet upon loss of PMF and the TM2 helix has been shown to exist in several conformations in artificial membranes [72]. The long C-terminal domain of D29 gp11 contains coiled-coil regions shown to be important in the oligomerization and lysis timing [55, 56]. Although the C-terminal domain is not highly charged (only 15 of 81 amino acids are KRHDE), amino acid substitutions show that several of the basic residues near the coiled-coil domain are critical to the ability of the holin to function [70]. Finally, the gp4 holin from Ms6 has a N-in-C-in topology and a small C-terminal domain of 18 amino acids with < 20% charged residues (Fig 3, panel C). Both of the TM helices do not have GxxxG motifs but do have high GA content and asymmetric polar residues that create a hydrophilic face. Gp4 is proposed to function as a pinholin since the TM1 helix has SAR-like properties similar to R^21^68 [31]. However, gp5 in Ms6 also has holin function and data show that gp4 can complement a λS mutant and this would suggest that it can form large holes [32]. Collectively, these results indicate that the presence of two TM helices cannot be used to classify how a putative holin will function in the lysis pathway and highlight the diversity in structural characteristics that exists in three well-studied holin proteins with two TM helices. The structural characteristics of these three well-studied holins was utilized to evaluate the diversity of the 15 *Gordonia* phage holin-like proteins that were predicted to have two TM helices.

**Fig 4:**
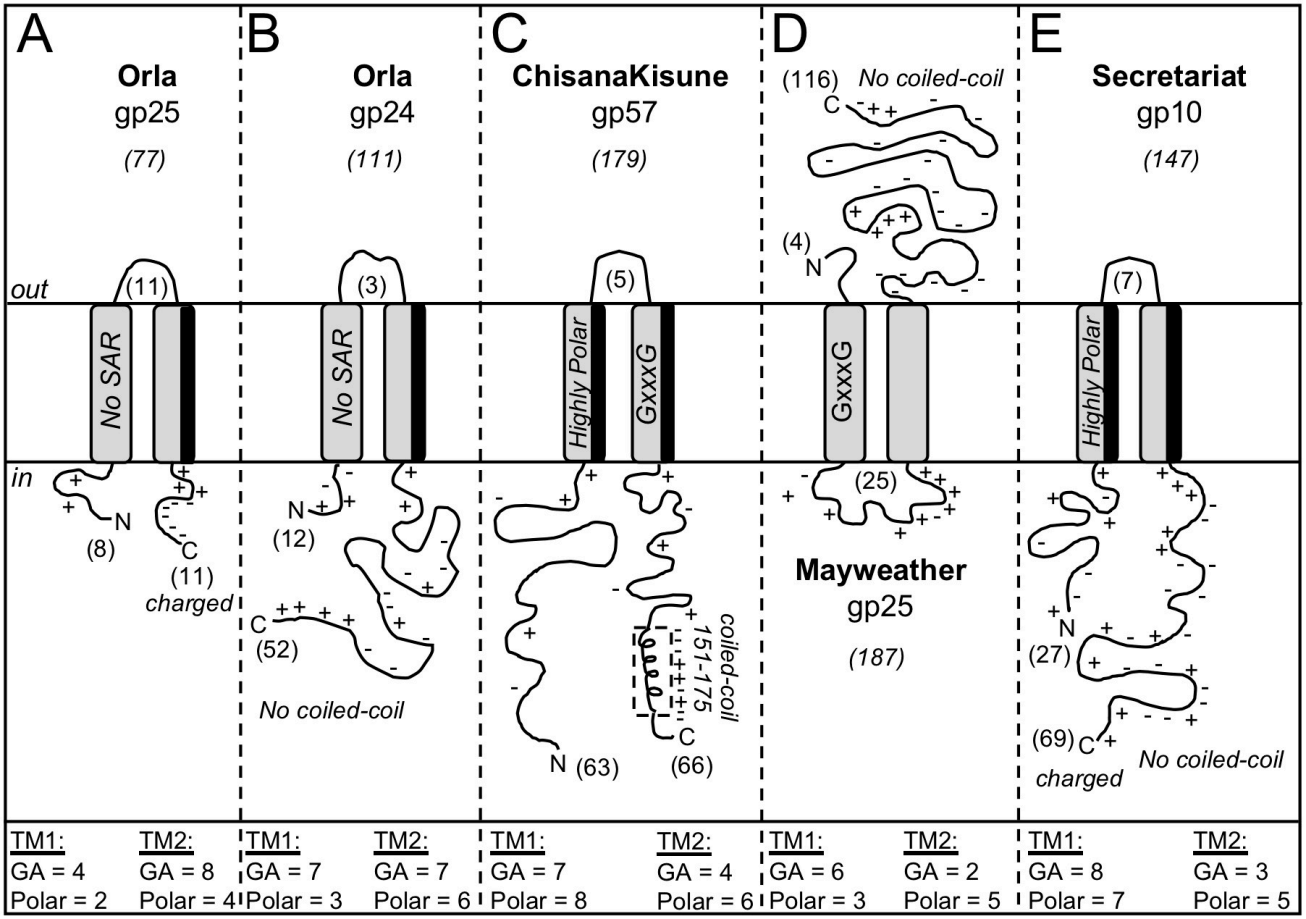
Topologies of proteins with two TM helices from *Gordonia* phages. TM helices are indicated by the gray areas spanning the membrane. TM helices with dark shading to one side indicate a hydrophilic face. Coiled-coil regions are shown as helix within a dashed box. Amino acid characteristics of TM1 and TM2 are shown in the bottom panel: GA = number of glycine and alanine residues in the helix; Polar = number of polar residues in the helix (STYQNDEKHR). N = N-terminal; C = C-terminal;+ = location of a positive residue (RKH);— = location of a negative residue (DE); numbers in parenthesis indicate the number of amino acids in a given domain or the size of the reference protein above the phage name. SAR = Signal arrest release sequence. GxxxG = the presence of a GxxxG region in the TM helix.

The fifteen two TM proteins have <20% amino acid identity, range of size from 72–187 amino acids and have a high level of diversity in the size of the N and C-terminal domains (Table 7). The 15 proteins are grouped into 10 different Phams and based on their size and structure, the proteins from the 10 Phams can be loosely grouped into five distinct topologies (Fig 4). The first group is the largest and contains the proteins from Suerte (gp21), Mayweather (gp24), Orla (gp25), Amok (gp21) and Jalebi (gp 36). Orla (gp25) is shown as the representative structure for these proteins (Fig 4, panel A). Proteins in this group are all <91 amino acids with the N-in-C-in topology, a small N-terminal region and a highly charged C-terminal domain with >45% KRHDE residues. Based on their size and general topology, this group most resembles the R^21^68 pinholin. However, the TM1 helix of these proteins is not predicted to have SAR function since there is not a high level of GA or polar residues (Table 7). In addition, none of these genes has a dual start motif that would generate a potential antiholin. The second topology contains Suerte (gp20) and Orla (gp24) and is shown in Fig 4, panel B. These are larger proteins of 111–126 amino acids with a short N-terminal and long much less charged C-terminal domain (<30% KRHDE). The TM1 helix is not highly polar but the TM2 helix is predicted to have an asymmetric distribution of polar residues that create a polar face to one side of the helix similar to TM2 of R^21^68. The large C-terminal regions appear to be unstructured and do not have a predicted coiled-coil domain like the gp11 from D29. The third topology includes ChisanaKitsune (gp61), DalanDe (gp56) and Clawz (gp66). These proteins range from 135–179 amino acids and have extended N and C terminal cytoplasmic domains of >40 amino acids (Fig 4, panel C). All have a highly polar TM1 helix with >64% polar residues and a TM2 helix that has one GxxxG motif and is also highly polar. Neither the N or C-terminal domain contain > 25% charged residues, although a coiled-coil domain is identified between amino acids 87–116 of gp61 from ChisanaKitsune. Interestingly, this region was not identified in gp56 from DalanDe even though both proteins are grouped in the same pham. In addition, the C-terminal domain of gp66 from Clawz has 60% amino acid similarity to ChisanaKitsune gp61, but in this protein the C-terminus is truncated and therefore missing the C-terminal 25 amino acids that contain the coiled-coil. This shows that even within the loose grouping on these holin-like proteins, there are additional structural differences that can be observed between the proteins.

**Table 7:** Characterization proteins with 2 TM Helices.

	Phage	Gene	Cluster	Pham designation from Table 5	Size of protein (amino acids)	N-terminal amino acids Orientation	N-term amino acids prior to TM1	N-term amino acids KRHDE	N-term amino acids % KRHDE	TM1 GxxxG Motif	TM1 Total AG residues	TM1 Total STYQNKRDE residues	TM2 GxxxG Motif	TM2 Total AG residues	TM2 Total STYQNKRDE residues	C-terminal amino acids Orientation	C-term amino acids after TM2	C-term amino acids KRHDE	C-term amino acids % KRHDE
*1*	Amok	gp21	CT	3	91	IN	21	6	29%	NO	4	5	NO	8	3	IN	18	7	39%
*2*	Suerte	gp22	CZ	9	168	OUT	6	0	0%	1	6	5	NO	4	9	OUT	105	33	31%
*3*	Secretariat	gp10	DJ	10	147	IN	27	7	26%	NO	8	7	NO	3	5	IN	69	26	38%
*4*	Jalebi	gp36	DV	12	89	IN	20	6	30%	NO	7	4	NO	8	9	IN	19	10	53%
*5*	Suerte	gp20	CZ	13	126	IN	13	4	31%	NO	5	5	NO	7	8	IN	65	19	29%
*6*	Mayweather	gp25	CT	14	187	OUT	4	0	0%	2	6	3	NO	2	5	OUT	116	28	24%
*7*	ChisanaKitsune	gp57	DQ	16	179	IN	63	6	10%	NO	7	8	1	4	6	IN	66	14	21%
*8*	DalanDe	gp56	DQ	16	172	IN	53	6	11%	NO	5	11	NO	5	7	IN	69	17	25%
*9*	Orla	gp24	CT	17	111	IN	13	4	31%	NO	7	3	NO	7	6	IN	52	16	31%
*10*	Clawz	gp66	CP	20	135	IN	45	5	11%	NO	5	9	1	7	7	IN	41	9	22%
*11*	Orla	gp26	CT	22	148	OUT	3	1	33%	1	7	4	NO	4	6	OUT	80	19	24%
*12*	DalanDe	gp58	DQ	25	101	IN	27	9	33%	NO	8	4	2	6	6	IN	16	5	31%
*13*	Mayweather	gp24	CT	33	78	IN	5	1	20%	NO	3	4	NO	5	5	IN	21	15	71%
*14*	Orla	gp25	CT	33	72	IN	8	2	25%	NO	4	2	1	8	4	IN	11	9	82%
*15*	Suerte	gp21	CZ	34	77	IN	14	5	36%	NO	4	4	NO	7	4	IN	12	7	58%
	*E*.*coli* Phage 21	R21	N/A	N/A	68	IN	3	2	67%	2	7	9	NO	5	5	IN	15	9	60%
	D29	gp11	A2	N/A	141	IN	8	3	38%	NO	6	5	NO	10	4	IN	81	15	19%
	Ms6	gp4	F1	N/A	78	IN	13	4	31%	NO	8	6	NO	6	5	IN	18	3	17%

The detailed information regarding the 15 proteins with two TM helices is provided. Proteins from the same Pham are shaded. The information for the holins from phage 21, D29 and Ms6 is provided for comparison.

The fourth topology has is predicted to have a unique N-out-C-out structure and includes Mayweather (gp25), Orla (gp26) and Suerte (gp22). These proteins contain 148–168 amino acids and all have a very small N-terminal region of 3–6 amino acids, a large highly charged cytoplasmic loop between the two TM helices and then an extensive extracellular C-terminal region with <30% KRHDE residues (Fig 4, panel D). The TM1 helix of all proteins has a high GA content and at least one GxxxG motif, but aside from the TM2 helix of Suerte, the TMs do not have a high proportion of hydrophilic amino acids (Table 7). These three proteins have <26% amino acid identity and no conserved residues were identified. However, the genes for these proteins are always found in the same location of the lysis cassette as the third gene of three consecutive holin-like genes directly downstream of the *lysA* (see the detailed lysis cassette schemes below). The last topology is gp10 from Secretariat (Fig 4, panel E). This protein is 145 amino acids with N-in-C-in topology and a large, highly charged C-terminal region (>38% KRHDE) that does not have a coiled-coil domain. The TM1 helix has both high GA and hydrophilic residues suggesting it may have SAR function, and both the TM1 and TM2 helix have the polar residues in an asymmetric orientation that creates polar faces. Finally, DumpTruck gp33 is also predicted to have 2 TM helices, but in this protein, the N-terminal TM helix is predicted to serve as a signal sequence (S1 Table). This scenario predicts that the one internal TM that would keep the protein embedded in the membrane in an N-out-C-in topology with the bulk of the protein localized outside the cell. Since gp33 is only 87 amino acids, the extracellular portion of the protein would be 54 amino acids and thus, this topology would be distinct from all of the single TM proteins described in the previous section (Table 6).

In summary, while the 15 proteins with two TM helices each have a few characteristics of the holins from phage 21, Ms6 and D29, they all have unique features not found in these proteins and do not show amino acid identity to these proteins. It is noteworthy that when any of the two TM genes are found in combinations with other two TM genes in the lysis cassette (Ex: Suerte, Mayweather, Orla, DalanDe), all of the two TM holin-like proteins expressed are never duplicates and are always from different Phams and have distinct topologies (see schematics described below).

#### Topologies and characterization of proteins with 3–4 TM helices

The holin proteins from phage λ (S105 and S107) and phage P2 (Y) have been classified as Type I and have 3 TM domains with an N-out-C-in topology [6, 7]. The LysA protein from phage P2 is the only holin functionally evaluated that has 4 TM domains with an N-in-C-in topology. It was striking therefore to find that only one of the 46 TM proteins from *Gordonia* phages is predicted to have three TM helices (KappaFarmdelta gp26), while 18 of TM proteins are predicted to have four TM helices. KappaFarmDelta gp26 has a predicted three TM topology that is N-out-C-in with a highly charged C-terminal domain (50% KRHDE) and no dual start motif. The TM1 helix has a similar GA and hydrophilic residue content to that of the TM1 helix of S105 of phage λ [17, 18]. Thus, KappaFarmDelta gp26 is the only TM protein in the current study that has the overall topology and structure of a canonical Type I holin.

Overall, the 18 *Gordonia* phages with four TM helices are grouped into 11 different Phams and all have the same N-in-C-in topology (Table 8). These TM proteins have much less variability in size compared to the proteins with only one or two TM domains and they range from 113 amino acids (VanLee gp29) to 164 amino acids (CaiB gp17). All the proteins have N and C-terminal cytoplasmic domains ranging from 5–30 amino acids and they also differ in the percentage of charged RKHDE residues. Table 8 shows that there are two proteins that do not have highly charged N or C-terminal regions with >30% RKHDE residues (KappaFarmDelta gp23 and LilPad gp32). Three proteins show a charged N-terminus (CaiB gp17 and gp18 and Mayweather gp23), and three show a charged C-terminus (Jalebi gp39, PokyPuppy gp36, DumpTruck gp34). The remaining 10 proteins show >30% charged residues in both the N and C-terminus. To determine if there were any conserved areas within the proteins that could help classify them from each other, one representative protein from all 10 Phams was aligned in Clustal Omega. The results were inconclusive as all proteins aligned with <22% identity and there were no regions of highly conserved amino acids. The analysis was repeated with each of the different TM helices that also revealed very low identity and no defined areas of conservation within any of the four TM helices. This is striking, given that the TM helices in holins appear to be very susceptible to amino acid mutations that render them ineffective [6, 7]. Thus, the lack of identity in any TM helix suggest that these proteins are in fact distinct from each other even though there are no obvious characteristics that differentiate them aside form the size and charges of the cytoplasmic domains. All of the four TM proteins are always found in lysis cassettes with at least two other TM proteins. In addition, as observed with the two TM genes, when more than one four TM gene is found in the lysis cassette, the expressed proteins are always different and never from the same Pham (see the Discussion in the schematics described below).

**Table 8:** Characterization proteins with 4 TM helices.

	Phage	Gene	Cluster	Pham designation from Table 5	Size amino acids	N-term amino acids	N-term ORI	N -term charges	N-term % charged	C-term amino acids	C-term ORI	C -term charges	C-term % charged
*1*	PokyPuppy	gp36	CS	**1**	180	28	IN	8	29%	25	IN	12	48%
*2*	DumpTruck	gp35	DV	**1**	162	30	IN	9	30%	19	IN	9	47%
*3*	CaiB	gp18	DR	**1**	153	19	IN	6	32%	12	IN	3	25%
*4*	LilyPad	gp34	DG	**1**	162	25	IN	8	32%	20	IN	10	50%
*5*	KappaFarmDelta	gp23	CV	**2**	150	24	IN	7	29%	17	IN	1	6%
*6*	Amok	gp22	CT	**7**	162	20	IN	9	45%	12	IN	7	58%
*7*	ChisanaKitsune	gp63	DQ	**18**	164	14	IN	5	36%	19	IN	6	32%
*8*	DalanDe	gp59	DQ	**18**	163	14	IN	5	36%	23	IN	8	35%
*9*	VanLee	gp30	sing	**21**	161	20	IN	7	35%	12	IN	8	67%
*10*	Jalebi	gp39	DV	**26**	128	5	IN	1	20%	12	IN	4	33%
*11*	LilyPad	gp32	DG	**28**	134	17	IN	3	18%	20	IN	4	20%
*12*	Mayweather	gp23	CT	**28**	117	15	IN	6	40%	5	IN	1	20%
*13*	CaiB	gp17	DR	**29**	164	23	IN	11	48%	14	IN	4	29%
*14*	DumpTruck	gp34	DV	**30**	150	19	IN	5	26%	10	IN	5	50%
*15*	PokyPuppy	gp35	CS	**30**	148	23	IN	8	35%	10	IN	5	50%
*16*	LilyPad	gp33	DG	**30**	148	20	IN	12	60%	8	IN	3	38%
*17*	CaiB	gp16	DR	**31**	133	29	IN	9	31%	9	IN	3	33%
*18*	VanLee	gp29	sing	**32**	113	8	IN	3	38%	6	IN	2	33%

The detailed information regarding the 18 proteins with 4 TM helices is provided. Proteins grouped to the same Phams are shaded.

### Determination of lysis cassette genome organization

All of the data from the previous sections was utilized to visualize the genomic organization of the putative lysis cassette for each of the 16 reference phages taking into account the location of the different TM genes in relation to the *lysA* and *lysB* genes. The analysis results in 16 distinct genomic orientations of the lysis cassette that has no defined pattern, but can be loosely grouped into seven different scenarios based on the number of TM genes and the location of the *lysB*. A key to all schematics is shown in Fig 1. Since Pham assignments in PhagesDB change as more genes are annotated it was pertinent to assign letters and numbers to the LysA and TM proteins to assist in identifying those within the schematics that are grouped to the same Pham. For the LysA proteins, each Pham was designated with a letter between A-J (Table 2). LysAs that are noted with the same letter are members of the same Pham. For the TM proteins a similar approach was used but with numbering between 1–34 (Table 5). The findings regarding each scenario are detailed below.

#### Scenario 1: A cassette with an endolysin associated with a single transmembrane gene and no lysB in the genome

**Fig 5:**
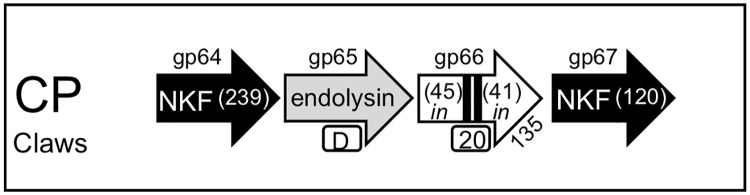
Genomic organization of a lysis cassette with a single transmembrane gene and no *lysB*. The genomic structure is show and the key to the schematic is presented in Fig 1. The direction of transcription of each gene is represented by the arrowhead.

The simplest lysis cassette found across all the phages in the current study was present in the CP cluster phage Clawz (Fig 5). This lysis cassette is the only one in the dataset that does not have a *lysB* and consists of a single endolysin (gp65) and a single two TM protein (gp66) directly downstream. These two genes are spanned by others that don’t have known function or TM domains and the cassette itself is localized downstream of the tape measure and minor tail genes. Gp66 does not fit the same topology as the canonical Type II holin since it is larger (135 amino acids) and also does not have a highly charged C-terminal domain. The TM1 helix of Clawz does contain a high percentage of polar residues but lacks a GxxxG motif found in the S^21^ pinholins. It is also relevant that both TM helices are located >40 amino acids from the N and C-terminus and this is much farther spaced from the protein termini than other the validated SAR domains of S^21^ and other proteins [17, 18]. Thus, it appears that gp66 is the only identifiable holin-like protein in the lysis cassette of Clawz and there is also no evidence for a dual start motif to tis gene. This minimal lysis cassette organization is similar to mycobacteriophage D29 where gp11 is the only TM protein in a lysis cassette between *lysA* and *lysB* (58). Since the lack of a *lysB* gene is not lethal to mycobacteriophages but been shown to impact lysis efficiency and overall phage fitness [29, 30], it’s intriguing that the endolysin in Clawz is the largest of all the LysA enzymes evaluated in this study and is predicted to have four distinct catalytic domains (Fig 2). Perhaps these multiple activities are important in overcoming the lack of *lysB* in this phage.

#### Scenario 2: A cassette with lysA associated with a single transmembrane gene and a predicted lysB at a distal location

**Fig 6:**
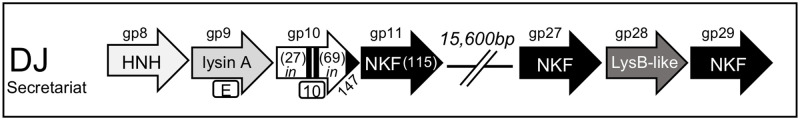
Genomic organization of a lysis cassette with a *lysA* and single transmembrane gene and a predicted *lysB* at distal location. The genomic structure is shown and the key to the schematic is presented in Fig 1. The direction of transcription of each gene is represented by the arrowhead.

The DJ cluster is one of the largest containing *G*. *rubripertincta* phages and Secretariat is representative of 15 other phages that all have genes from the same phams within the lysis cassette. This cassette is simple like Clawz and also contains a single *lysA* and a single downstream two TM gene (Fig 6). In Secretariat, the cassette is localized significantly upstream of the tape measure and other structural genes unlike most other *Gordonia* phages in this study. Secretariat has a predicted *lysB* (gp28), but it is not associated with the lysis cassette and is located ~15,000bp downstream from the *lysA*. As described previously, gp10 has a unique topology and does not fit with the canonical Type II holins since it does not have a dual start motif, is nearly twice as large as S^21^68 and has much more extensive N and C-terminal domains (Figs 3 and 4). The TM1 helix of gp11 does contain 32% polar residues and has a high GA content (but no GxxxG motif), making it possible that it may act as a SAR. However, unlike the SAR in S^21^68 that is at the very N-terminal region of the protein, the TM1 of gp10 is localized 27 amino acids from the N-terminus that also has numerous charged residues that may influence how the TM1 helix would be oriented. Thus, DJ phages appear to express a single holin-like protein from a defined lysis cassette.

#### Scenario 3: A cassette of three transmembrane genes flanked by lysA and lysB

**Fig 7:**
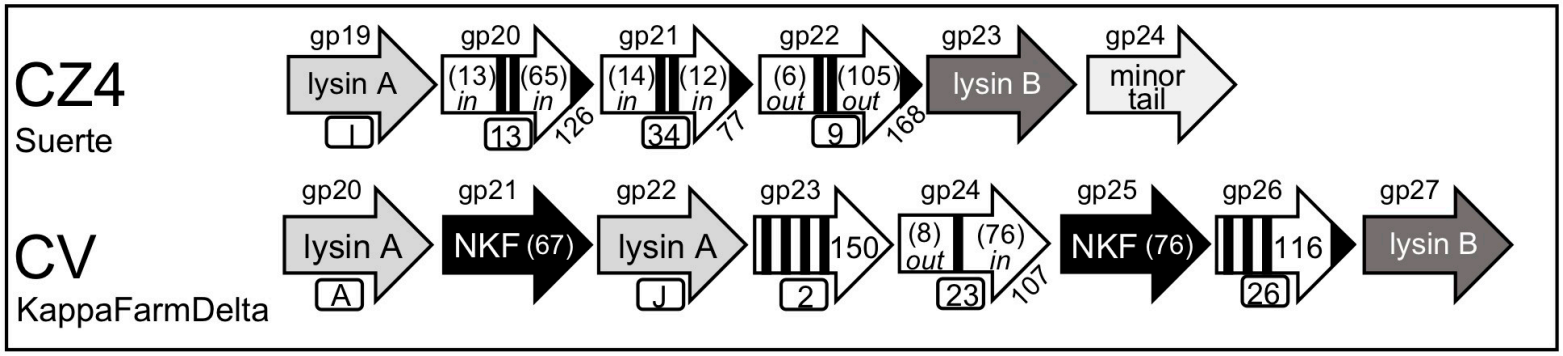
Genomic organization lysis cassettes with a three transmembrane genes flanked by *lysA* and *lysB*. The genomic structure is shown and the key to the schematic is presented in Fig 1. The direction of transcription of each gene is represented by the arrowhead.

Suerte (cluster CZ4) and KappaFarmDelta (CV) have multi-gene lysis cassettes that contain both *lysA* and *lysB* genes and these flank three genes that encode proteins with TM helices (Fig 7). These lysis cassettes represent the first that have been formally described to contain more than two TM proteins. The *lysA* genes in these phages each encodes enzymes with different activities toward the PG (Fig 2). Both lysis cassettes are found directly downstream of the tape measure and other structural genes. Suerte represents three other CZ4 cluster phages while KappaFarmDelta is the only phage in CV that infects *G*. *rubripertincta*. Although the three TM proteins in Suerte all have two domains, each TM protein has a different size, is from a different Pham, and has a distinct topology (Table 7). Thus, this finding suggests that each gene is unique and is not simply a product of a gene duplication event. In contrast, the TM genes in KappaFarmDelta encode proteins predicted to have one, three, or four TM helices and each also has a distinct topology. The KappaFarmDelta cassette also includes two other genes that encode small proteins don’t have known function or TM helices (gp21 and gp25) that may also be involved in the lysis pathway due to their location within the cassette. The finding that there are three distinct TM proteins expressed from each lysis cassette suggests that each may have a defined function in the lysis pathway. This could include being a timer, a holin or an antiholin. However, since the holin and antiholin proteins most studied are encoded from the same gene using a dual start motif [6, 7], it is not possible to assign holin and antiholin function based on topologies without functional studies.

#### Scenario 4A: A cassette with lysA associated with three transmembrane genes and a predicted lysB at a distal location

**Fig 8:**
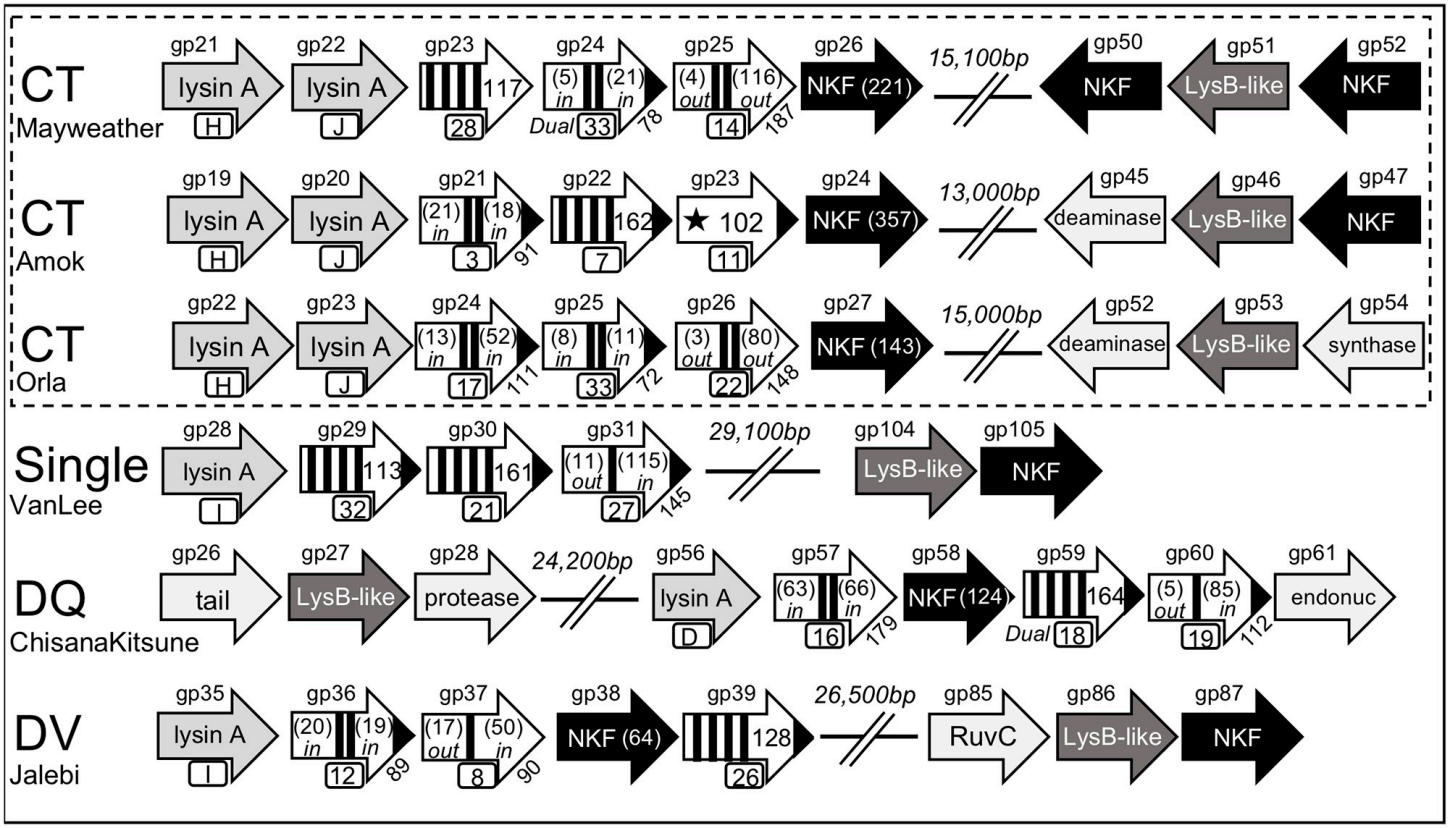
Genomic organization of lysis cassettes with a *lysA* and three transmembrane genes and a predicted *lysB* at a distal location. The genomic structure is shown and the key to the schematic is presented in Fig 1. The direction of transcription of each gene is represented by the arrowhead. Dual = Gene has a possible dual start motif and contains a basic amino acid between the two start codons.

The fourth scenario found in the *Gordonia* phages is a lysis cassette that contains a *lysA* and three TM genes that are localized at a distance from a predicted *lysB* (Fig 8). Within this scenario there are six distinct organizations that represent a total of 40 phages. The CT cluster contains 23 phages and all have split *lysA* genes that are grouped to the same Phams and express proteins with the same activities (Table 2). The *lysA* genes in CT phages are all localized downstream of the tape measure and other structural genes. The predicted *lysB* genes are localized 13,000–15,000bp further downstream of the *lysA*. However, despite having the same *lysA* genes, the 23 CT phages show nucleotide divergence that occur within the last 150 nucleotides of second *lysA* gene and extends directly downstream for ~11,000bp. This results in variation in the gene organizations downstream of the *lysA* and the presence of three distinct sets of holin-like genes within the CT cluster of which only one of the nine genes is conserved (Fig 8). The first scenario, typified by Mayweather and found in eight other CT phages, is a linear stretch of three genes that encode proteins with four, two and two TM helices. The second scenario typified by Amok and found in 11 other CT phages contains genes that encode proteins with two and four TM helices that are distinct from those in Mayweather. In addition, Amok has a third gene in the cassette that encodes a protein with single N-terminal TM helix that is predicted by the majority of the analysis programs to be a signal sequence (See S1 Table). Thus, this predicts that gp23 is a secreted protein that may or may not have a function in the lysis pathway. Finally, Orla has an organization found in four other CT phages with a linear stretch of three genes that all encode two TM proteins (Fig 7). Interestingly, based on protein topology, the linear order of the three encoded TM proteins is exactly the same in Orla and Suerte but only the second TM gene of these lysis cassettes are from the same Pham (Table 7). It is also noteworthy that the third TM gene in the linear arrangement of both cassettes is not from the same Pham but encodes the unique two TM protein with the novel N-out-C-out topology (Fig 4). Thus, in spite of being from the same cluster, the CT phages show dramatic diversity in the three holin-like proteins that would be predicted to be encoded from the lysis cassette (Fig 7).

The singleton phage VanLee has an organization with a single *lysA* within a lysis cassette downstream of the tape measure and a predicted *lysB* found 29,000bp further downstream. Like the CT phages, VanLee has three genes in a direct linear organization but these express proteins with four, four, and one TM helices (Fig 8). VanLee gp29 and gp30 each have four TM domains but are not in the same Pham and are 113 and 161 amino acids, respectively (Table 8). While each of these proteins have >33% changed N and C-terminal domains, the C-terminal domain of gp29 is nearly 70% changed with an end sequence of RDDREARKKNAT, making it clearly different from gp30. ChisanaKitsune represents three other DQ phages and these phages have a *lysB* gene 24,000bp upstream of a lysis cassette that contains the very large 1,100 amino acid LysA with four domains that is also found in Clawz (Table 2 and Fig 2). Downstream of the *lysA* are a series of three TM proteins with the first two separated by a small NKF gene (Fig 8). The expressed TM proteins contain two, four, and one TM helix and none of these are in the same Phams as the TM proteins from the CT phages. Interestingly, the 14 phages from the DV cluster, typified by Jalebi, also have a lysis cassette that expresses proteins with one, two, and four TM helices (Fig 8). However, in the DV phages, the genes are organized in a two, one, and four orientation where the last two genes are separated by a small NKF gene. Despite the fact that the DV and DQ phages express three proteins with similar numbers of TM helices, none of the proteins are grouped to the same Phams and both sets of the one TM and two TM proteins have distinct topologies. Thus, the six lysis cassettes each have a unique gene organization with four different *lysA* genes and 17 of the 18 encoded TM proteins grouped to distinct Phams (Figs 7 and 8).

#### Scenario 4B: A cassette with lysA associated with four transmembrane genes and a predicted lysB at a distal location

**Fig 9:**
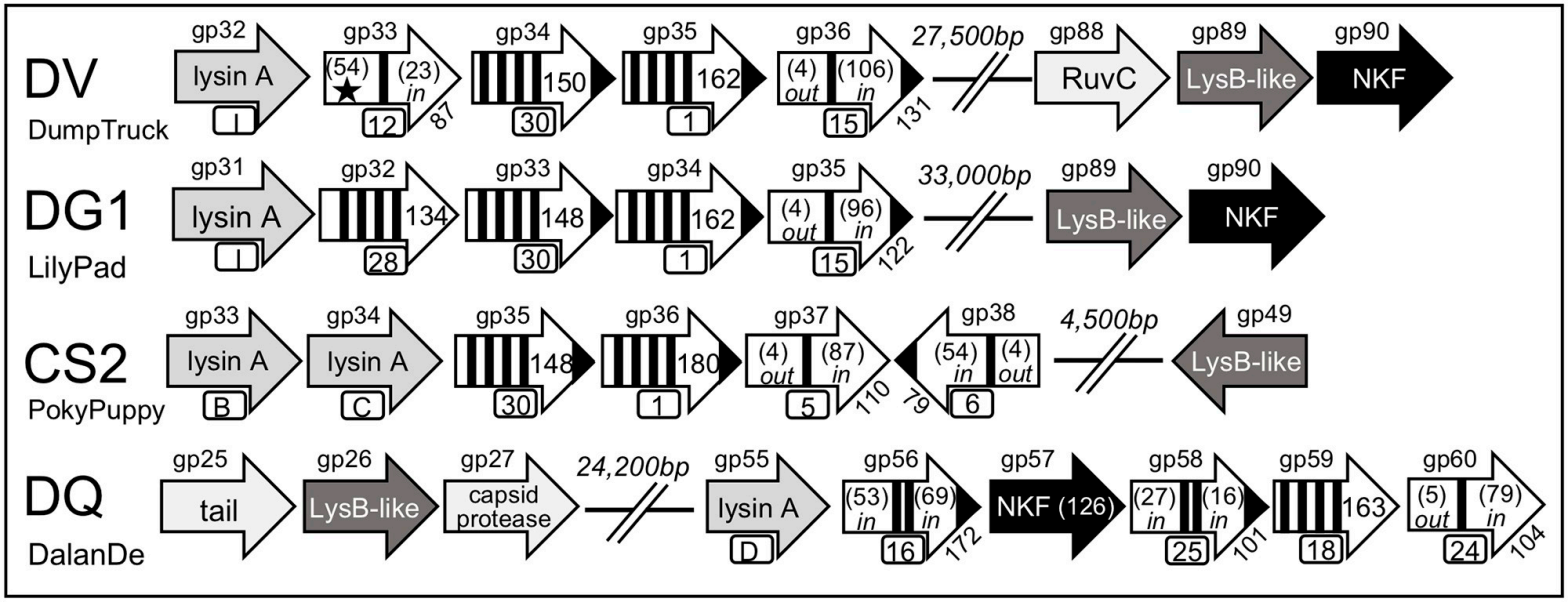
Genomic organization a lysis cassettes with a *lysA* and four transmembrane genes and a predicted *lysB* at a distal location. The genomic structure is shown and the key to the schematic is presented in Fig 1. The direction of transcription of each gene is represented by the arrowhead.

The finding with the fifth scenario is four predicted TM genes downstream of one or more *lysA* genes and the *lysB* present in the genome at a distal location (Fig 9). All four cassettes in this scenario have a distinct gene organization and contain three different l*ysA* genes, although DumpTruck (cluster DV) and LilyPad (cluster DG1) share four of the five genes in the lysis cassette. In this regard, both of these phages express a single LysA from the same Pham and only diverge at the first of the four TM genes. In DumpTruck, the first TM protein predicted by most programs and HHpred has a signal sequence and also one internal TM helix that would render this a one TM proteins with an N-out-C-in topology. In contrast, the first gene downstream of the *lysA* in LilyPad encodes a larger four TM protein that is also the first TM gene found in Mayweather (gp23, Fig 8). The first TM gene in DumpTruck and LilyPad is followed by the same three TM genes that encode proteins with four, four, and one TM helix. Interestingly, PokyPuppy also has two of the four TM genes found in DumpTruck and LilyPad in its lysis cassette (gp35 and gp36). However, PokyPuppy has two *lysA* genes and the final two TM genes encode proteins with a single TM helix. The fourth TM gene identified in PokyPuppy (gp38) is unique in that it is the only TM gene in this study that is in a reverse orientation to the lysis cassette. Thus, this gene would not be transcribed in an operon with the other genes in the cassette. However, unlike all of the other scenarios where a distally localized *lysB* is found 13,000–30,000bp from the lysis cassette, the *lysB* in PokyPuppy (gp49) is only 4,000bp from the lysis cassette and in the same reverse orientation as gp38 (Fig 9). Thus, it is likely that the *lysB* and gp38 would have the same temporal expression.

DalanDe is the fourth phage with four TM genes in the lysis cassette. gp56 and gp59 express TM proteins with two and four TM helices, respectively, and both genes are also found in the lysis cassette of the other DV phage, ChisanaKitsune (Fig 8). However, DalanDe contains two additional TM genes within the lysis cassette that encode proteins with two TM helices (gp58) and one TM helix (gp60). What is interesting about these two genes is that they are that are fully unique to DalanDe and are the only ones in the study that do not have additional Pham members (Table 4). That these orpham genes are integrated between the others added further support that phages acquire individual genes to established lysis cassettes.

#### Scenario 5: A cassette with lysA and lysB and four transmembrane proteins identified at a distal location

**Fig 10:**
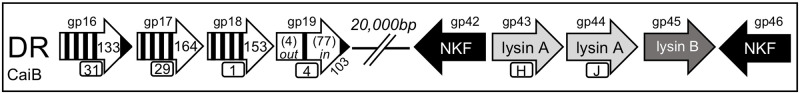
Genomic organization a lysis cassettes with a *lysA* and *lysB* in the same location and four transmembrane genes at a distal location. The genomic structure is shown and the key to the schematic is presented in Fig 1. The direction of transcription of the gene is represented by the arrowhead. The identity of each protein is shown above the arrow.

CaiB is a DR cluster phage and represents five other phages with the same genome organization. CaiB has a split *lysA* in the same Phams as CT phages Amok, Mayweather and Orla, but the *lysA* genes are found in a three-gene forward cassette with *lysB* (Fig 10). This lysis cassette is spanned upstream by a series of three reverse genes and downstream by a series of 14 reverse genes with various enzymatic activities (Fig 10). Neither region contains any TM genes. Although it has been shown that SAR sequences [6, 7] or secretion system [23, 33] can assist endolysins from exiting the IM in a holin-independent manner, there are no phages that have been formally characterized that function without some type of holin-like protein to control the triggering event. The LysA and LysB proteins from CaiB have no evidence of SAR domains. Thus, the full genome of CaiB was evaluated to determine if TM proteins could be identified at distal locations to the lysins. A series of four TM proteins with overlapping open reading frames was identified between gp16-19, that are also present in all other DR phages. This “holin cassette” of TM genes resembles the orientations discussed in previous sections and the genes encode proteins with four, four, four, and one TM helix. There are several pieces of evidence that support a holin function for these proteins. First, the Phams for each of these proteins contain >114 members with the Pham of gp16 having over 100 members at the time of this report. Second, the analysis of the location genes from the Pham members, show that many are found associated with a lysis cassette that contains a *lysA* gene. For example, homologs to CaiB gp17 and gp19 are found in the previously described lysis cassettes for PokyPuppy, LilyPad and DumpTruck (Fig 8). Finally, this type genomic organization with a cassette of enodolysin genes and a separate distal cassette of holin-like genes is observed in several clusters of phages that infect *M*. *smegmatis* or *G*. *terrae*. AN, B3 and B5 cluster phages all have a four gene cassette of TM genes, while B1 and B4 cluster phages have a cassette with two TM genes located ~20,000bp upstream, of the endolysin genes. Thus, these results clearly show that holin-like genes are not always localized within a linear cassette with the endolysin or *lysA* adding a further layer of complexity to the genomic organization of the lysis pathway genes.

#### Scenario 6: A cassette with lysA and lysB and no holin-like genes within the entire genome

**Fig 11:**
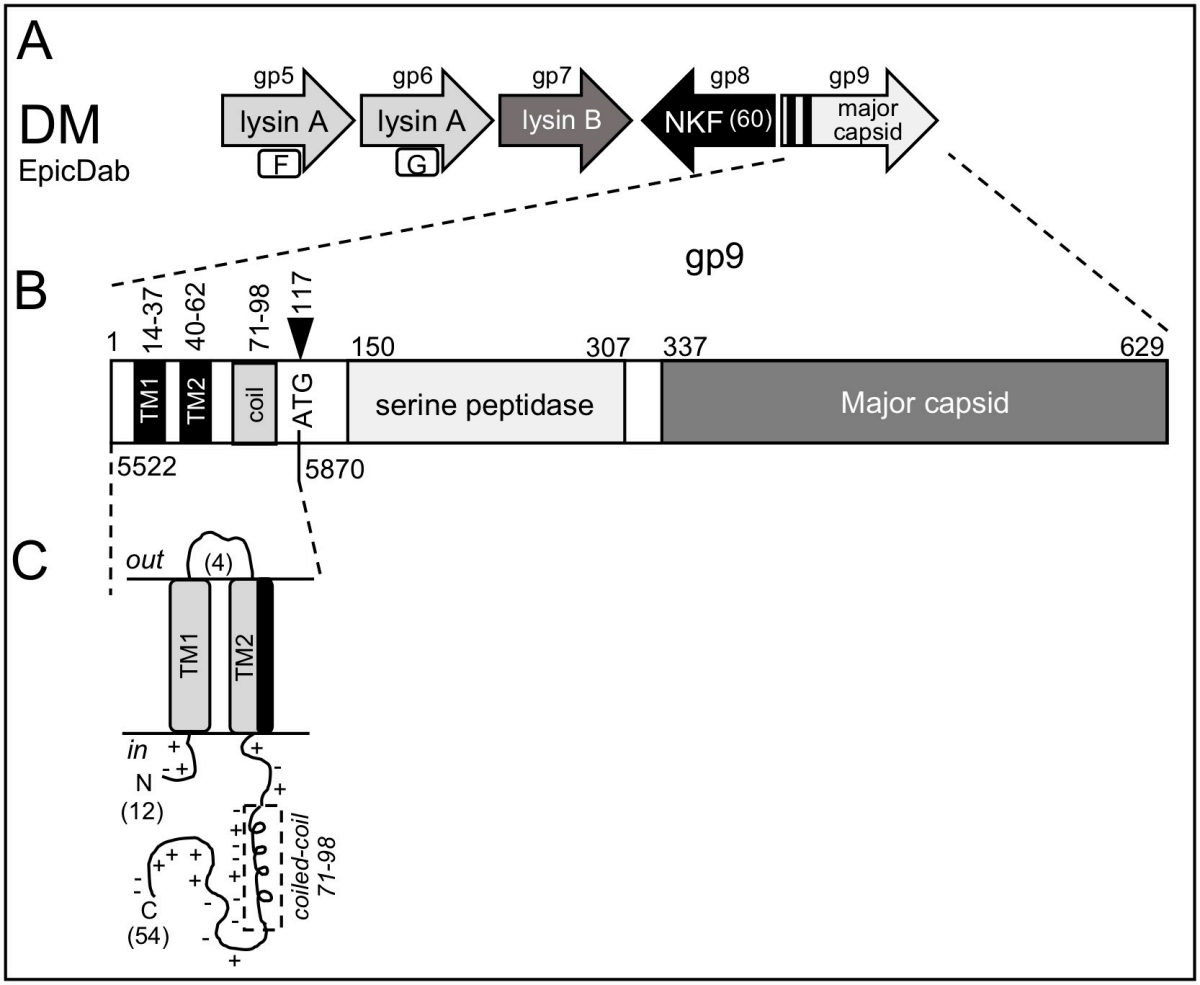
Genomic organization a lysis cassettes with a *lysA* and *lysB* in the same location and no holin like genes. A. The genomic structure is shown and the key to the schematic is presented in Fig 1. The direction of transcription of each gene is represented by the arrowhead. B. Structure of the major capsid protease fusion protein. All domains identified are shown with amino acid numbers on the top. The ATG start codon at nucleotide 5870 is shown by the arrowhead. C. Predicted structure of the protein region between amino acids 1–116 if it was liberated from the full-length protein. TM regions are indicated by the gray areas spanning the membrane. TM regions with dark shading to one side indicate a hydrophilic face. Coiled-coil region is shown in dashed box. N = N-terminal; C = C-terminal;+ = location of a positive residue (RKH);— = location of a negative residue (DE); numbers in parenthesis indicate residues.

The last scenario is represented by the microphage EpicDab. This is a DM cluster phage with a genome that is only 16,658bp and is significantly smaller than any of the other reference phages (Table 1). EpicDab is in the DM cluster with SallySpecial and Emperor, but is the only phage that infects *G*. *rubripertincta* as the host. EpicDab has 26 protein coding genes of which nine encode structural proteins and eight encode various enzymes including LysA (gp5 and gp6) and LysB (gp7). EpicDab has a lysis cassette with the two *lysA* and *lysB* genes (Fig 11, panel A), however, there are no putative holin-like TM genes within the cassette or within the genome. Detailed analysis the nine NKF genes for TM helices revealed that all were predicted to be soluble proteins. The analysis of all forward and reverse open reading frames that were between the *lysB* (gp7) and major capsid (gp9) revealed no proteins with TM helices. However, analysis of the major capsid protein (gp9) showed that there were two TM domains predicted in the N-terminal 62 amino acids and a putative coiled-coil that is not associated with these helices between amino acids 71–98. The two TM helices are predicted in all TM analysis programs and also by HHpred. It has been established that the major capsid protein of phage HK97 contains a cleaved N-terminal “delta” domain that functions as a scaffolding protein, and is released from the final virion capsid. The HK97 delta domain contains coiled-coils but does not have defined TM helices [74–76]. Thus, it is possible that the N-terminal portion of EpicDab gp9 may be liberated from the final major capsid protein and function in a similar manner to the delta domain in head assembly.

The entire ORF of gp9 starts at base-pair 5,081 and overlaps to *lysB* gp7. However the annotated start is called at base-pair 5,522 to account for a predicted NKF reverse gene (gp8) that runs from base-pair 5,546–5,264. This results in gp9 having a 1,887bp ORF that encodes a 629 amino acid protein. HHpred analysis of gp9 reveals that there are predicted pfam serine peptidase alignments to a domain that spans amino acids 150–307, well after the N-terminal domain with the TM helices (Fig 11, panel B). The major capsid alignments are to a domain that spans amino acids 337–639 (Fig 11, panel B) and this is consistent with the average size of an actinobacteriophage major capsid protein that typically ranges from 300–350 amino acids [34]. In gp9 there is an ATG codon at nucleotide 5,870 that immediately precedes the protease and major capsid domains identified by HHpred. What is intriguing about the N-terminal domain of EpicDab gp9 in comparison to the delta domain of the major capsid of HK97, is that if the 116 amino acid N-terminal domain is excised and analyzed through the various TM prediction programs, the protein is predicted to have and N-in-C-in topology with a 54 amino acid C-terminal cytoplasmic domain that is highly charged and is also predicted to have a coiled-coil domain outside of the two TM helices (Fig 11, panel C). The predicted topology of this holin-like protein is remarkedly similar to that of the D29 gp11 holin (Fig 3B). Analysis of the gp9 protein sequence using a protease prediction tool [77], shows that there is a predicted serine protease (chymotrypsin) site at amino acid 145 just before the protease domain, although this is not the only site predicted to be cut by program. Thus, even if the domain serves to assist with head assembly, the amount of proteins produced and the timing of its liberation from the full-length major capsid would place it temporally at a point in the lytic cycle to also function in a “holin-like” role. Currently, the liberation of a holin from another functional protein via a protease has not been described, but clearly the lysis pathway genes has been shown to utilize dual start motifs to make multiple holins and antiholins [6, 7, 78], internal start sites to make smaller lysins [23, 24] and overlapping alternate reading frames to make large and small spanins with different amino acids sequences [14–16]. The intriguing findings in EpicDab suggest that this may constitute a novel mechanism for generating a holin-like protein in a phage with a restricted size genome.

## Conclusions and implications

A comprehensive bioinformatic approach has been utilized to identify the genes within the putative lysis cassette and characterize the encoded proteins from a collection of 77 phages that all infect *Gordonia rubripertincta* NRLL B-16540. This represents the first such analysis from a large set of phages that also includes a detailed evaluation of the membrane or holin-like proteins that are encoded by genes in the lysis cassette.

### Key findings from phages that infect *G*. *rubripertincta*

#### Diversity of LysA enymes

All 16 reference phages contained genes encoding an endolysin equivalent protein (LysA) and is predicted to target the PG layer. The identification of this gene was used to establish the location of the putative lysis cassette in the 16 *Gordonia* reference phages. Although the LysA enzymes from phages that infect *G*. *rubripertincta*, appear to contain many of the same domains found in the LysA from mycobacteriophages, they differ from these phages in several important ways. First, the majority of the LysA enzymes evaluated are smaller (175–360 amino acids) and do not have multiple enzymatic domain that target different areas of the PG. Thus, these LysA enzymes more resemble the smaller, single domain endolysins found in T4, T5, T7 and lambda phages that all infect E. hosts [9]. Only phages Clawz and DalanDe diverge from the other *Gordonia* phages with regard to the size of the LysA as these phages express a protein of >1000 amino acids that is even larger than those found in myocbacteriophages. Second, several of the *Gordonia* phages express two distinct LysA proteins, each that is < 300 amino acids and with a single enzymatic domain (like the lambda endolysins noted above). These split *lysA* genes express proteins with varying combinations of enzyme activities. The contribution of a single LysA activity to the PG or split genes with distinct activities to the lysis pathway and phage fitness is unknown. However, the split *lysA* genes may be an evolving feature in *Gordonia* since this organization has also been detected in phages that infect other *Gordonia* strains [25].

#### Diversity of LysB enzymes

Since the Gram-positive *Gordonia* bacteria have an OM rich in mycolic acid [19–21], a putative *lysB* gene that encodes a protein with predicted activity toward the mycolic acids in the OM was also identified in 15 of the reference phages. It is speculated that the LysB enzymes represent the third component of the proposed three-step lysis pathway [6, 7], and are the functional equivalents to the spannins that disrupt the OM and are expressed in phages that infect Gram-negative bacterial hosts [6, 7, 27–29]. The identified LysB proteins from the *Gordonia* phages has strong HHpred hits to the crystal structure of LysB that has been evaluated in wet lab experiments from mycobacteriophage D29 [28]. However, in the mycobacteriophages, the *lysB* genes are typically found very near the *lysA* in a putative lysis cassette [22, 23]. Although distally located *lysB* genes have been bioinformatically identified in other *Gordonia* phages [25], these proteins have not been characterized enzymatically. Thus, it’s possible that the distally located *lysB* is not expressing an enzyme involved in the lysis pathways since the temporal expression of the protein would be divergent from the genes in the putative lysis cassette. Alternatively, the distal genomic organization of the identified *lysB* may reflect the non-essential role of this protein in the lysis pathway [29, 30], and wet lab experiments will be required to validate whether phages that contain *lysA* and *lysB* in a defined lysis cassette have a fitness advantage over those that express the gene in a distal location.

#### Diversity of holin-like proteins

A wide variety of genes that encode holin-like proteins containing one-four TM helices were identified immediately downstream of the *lysA* in all but two of the 16 reference phages. In all, 46 genes that encode 32 distinct membrane proteins were identified with the majority of the reference phages having three or four of these holin-like genes. Thus, one of the key findings of this report is the striking diversity regarding the organization of these different genes within the lysis cassette and the identification of up to four holin-like genes within the lysis cassette. This type of organization has not been described in phages that infect Gram-negative hosts [6, 7]. There is no pattern to the holin-like gene organizations and different combinations of the *lysA*, *lysB* and holin-like genes are observed not only in genetically diverse phages, but also in phages that are grouped to the same cluster based on gene content. While all phages have the same *lysB* gene and several groups share the same *lysA*, only 12 of the 46 distinct holin-like genes are shared between any of the reference phages. This results in 16 different lysis cassette genome organizations within the population of 77 *Gordonia* phages evaluated. The level of diversity in the linear gene organization and changes to one or more genes within the ~3,000bp lysis cassettes suggest that the cassette itself is unlikely to be acquired by a phage as a single multi-gene module. Instead, it appears that phages acquire and lose individual holin-like genes within the lysis cassettes.

The striking diversity observed within the lysis cassette is mostly due to the number and topology of the different membrane or holin-like proteins identified. These holin-proteins have between one-four TM helices and although they have some of the characteristics of the Type I, Type II and Type III holins from phages that infect Gram-negative hosts [6, 7], the majority of the proteins have distinct structures in regard to: 1) size, 2) protein topology, 3) size of the extracellular and cytoplasmic domains, 4) number of charged residues in the cytoplasmic domains and 5) number of GA and hydrophilic amino acid residues in the various TM helices. Due to the large number of holin-like genes identified, it is noteworthy that two of the reference phages contained lysis cassettes with *lysA* that did not have associated holin-like genes. The DM cluster phage EpicDab does not have a defined holin-like gene in the entire genome. However, EpicDab expresses an exceptionally large major capsid protein with an N-terminal domain of 116 amino acids that has two well defined TM helices and may be cleaved from the native protein. Since EpicDab is a microphage with only 26 genes and a genome of 16,658bp, this novel gene organization may have evolved due to the compactness of the EpicDab genome. The DR phages typified by CaiB also do not have a lysis cassette that contains both holin-like genes and *lysA*. However, these phages appear to have acquired a forward lysis cassette that contains two *lysA* and one *lysB* and a second cassette that contains four consecutive holin-like genes in a distinct operon-like region. The validity that these distal genes are in fact holin-like genes is supported by the finding that homologs of the expressed proteins are found in defined lysis cassettes with *lysA* in phages that infect other strains of Gram-positive bacteria. This finding is significant as it highlights that there are alternate organizations to the canonical lysis cassette and the linear arrangement of a *lysA* and holin-like genes may not always be present. A non-linear organization of the lysis genes is also found in the T4 phages (68, 69). It is also noteworthy that the small 1,600bp holin cassette in the DR phages is not found as an intact module in any of the other phages. Yet the third gene of the holin-like cassette in CaiB (gp18), is present within a lysis cassette that contains a *lysA* in three of the *Gordonia* reference phages (DumpTruck, LilyPad and PokyPuppy).

### Implications to established phage lysis models and lysis cassette evolution

Currently, all of the well-studied lysis models propose an interaction of no more than two holin-like proteins and in the case of λ and P2 the two proteins are actually generated from a single gene due to a dual start motif [6, 7]. In addition, the Gram-positive phage Ms6 has only two defined holin genes each expressing a single protein while phage D29 appears to function with a single holin encoded by gp11 [30–32, 67–72]. Thus, it is unprecedented to find lysis cassettes that contain up to four holin-like genes. In this regard, it is important to note that the current study shows that when more than two holin-like genes are present in the *Gordonia* phage lysis cassette, each of the holin-like proteins that are predicted to be expressed have a distinct topology and are unique, even if they have the same number of TM helices. Thus, the multiple genes are not simply “duplicated”, and the distinct sizes and topologies of the protein products suggest that each may impart a unique functionality. As described previously, holins function as timer to the lysis pathway and the Type I and Type III holins also function in the generation of large holes that allow the release of the endolysins to metabolize the cell wall components. The type II pinholins only serve as a timer and require the endolysins to be exported from the cell via a SAR. Since SAR regions were not identified in any of the LysA or LysB enzymes, and the *Gordonia* phages in this report do not appear to express a homolog to the gp1 secretory protein from phage Ms6 [30–32], it is hypothesized that the phages from the current study generate at least one canonical holin that allows the movement of the LysA and LysB through the membrane. Thus, one explanation for the expression of multiple holin-like proteins is that each may function in *only one* aspect of the lysis pathway: either hole formation or timing, not both. In this context, it’s also possible a protein may function as either a holin or antiholin. Thus, the interaction of all of the holin-like proteins would be important in the timing and regulation of the lysis pathway. Unfortunately, the best characterized antiholins are derived from the same gene as the holin using a dual start motif [6, 7]. Thus, there is minimal amino acid or topological diversity to distinguish the two proteins aside from a single charged residue in the N-terminal region that appears to play key role in how the TM1 domains are oriented before and after the loss of the PMF at triggering. The data from the current report show that the lysis cassettes that contain multiple holin-like genes always encode very different proteins with distinct topologies. Therefore, any prediction of holin or antiholin function will require future wet lab work.

The finding that the majority of *Gordonia* phages genomes contain a cassette with three or more distinct holin-like genes suggest the intriguing possibility that phages acquire multiple holin-like genes to enhance phage fitness and the ability of the phage to diversify the lysis pathway possibly in response to the evolution of the bacteria. The recent report showing that bacteria are evolving host defense that can target the lysis pathway [8] imply that phages would require mechanism to overcome this problem. Support to this idea is provided by recent work on gp11 from D29. This work shows that modifications to the 81 amino acid cytoplasmic C-terminal region can result in a holin that exhibits increased levels of cytotoxicity to both *E*. *coli* and *M*. *smegmatis* when the protein is induced from a plasmid construct [70]. While these studies were not performed in the context of a phage infection, they suggest that changes to the holin topology can generate a protein with different functional characteristics that might impact phage fitness and lytic activity. All of the holin-like proteins described in this report have naturally occurring differences in their cytoplasmic and extracellular domains. Thus, it is possible that the *Gordonia* phages that express multiple holin-like genes, a LysA with several activities and also a LysB may a selective advantage regarding their fitness. In summary, this study provides a framework for the bioinformatic analysis of the putative lysis cassette and characterization of holin-like proteins in phages that infect Gram-positive bacteria. Further wet lab evaluation of these novel lysis cassettes may reveal new lysis pathway models and provide insights into phage fitness, evasion of host defense systems and insights into the genetic engineering of holins with enhanced functionalities.

## Supporting information

S1 Table Detailed information for all TM proteins. The 46 TM proteins as listed in order of consensus prediction of total TM helices. The number of predicted TM helices for each TM prediction program are listed. The final column presents notes related to the consensus prediction.
(XLSX)

S2 Table Detailed analysis of HHpred hits for LysB proteins. The refence phage and LysB gene number is presented. The first 5 HHpred PDB hits for all LysB proteins are presented by PDB number. The description of each PDB is presented on the bottom of the table.
(XLSX)
